# Anti-inflammatory effects of *para*-quinone methide derivatives on ulcerative colitis

**DOI:** 10.3389/fphar.2024.1474678

**Published:** 2024-10-29

**Authors:** Yue Qiu, Xin Li, Xu Zhang, Xiaotong Wang, Xuekun Wang, Jie Yang, Guoyun Liu

**Affiliations:** ^1^ State Key Laboratory for Macromolecule Drugs and Large-Scale Manufacturing, School of Pharmaceutical Sciences, Liaocheng University, Liaocheng, China; ^2^ Liaocheng Key Laboratory of Quality Control and Pharmacodynamic Evaluation of Ganoderma Lucidum, Liaocheng University, Liaocheng, Shandong, China

**Keywords:** anti-inflammatory, ulcerative colitis, para-quinone methides, oxidative stress, apoptosis

## Abstract

A series of *para*-quinone methide derivatives were evaluated their anti-inflammatory activity. Through the screening of the lipopolysaccharide (LPS)-induced inflammatory cell model in Raw264.7 cells, it was found that the inhibitory activity of *meta*-substituted derivatives on NO production was superior to that of *ortho*- and *para*-substituted derivatives. Among them, in the inflammatory cell model, the *meta*-trifluoromethyl substituted *para*-quinone methide derivative **1i** had the best activity in inhibiting LPS-induced excess generation of NO. And **1i** could effectively inhibit the increase of ROS in inflammatory cells, the expression of iNOS related to the production of NO, and the expressions of inflammation related initiating protein TLR4, pro-inflammatory cytokines IL-6 and TNF-α, inflammasome NLRP3 and Caspase1. In the dextran sulfate sodium (DSS)-induced ulcerative colitis (UC) mouse model, the active derivative **1i** could inhibit DSS-induced colon shortening, and reverse DSS-induced pathological changes in colon tissue, such as inflammatory infiltration, structural destruction and crypt disappearance. **1i** could effectively inhibit oxidative stress, inflammation and apoptosis in UC mice. Moreover, through the determination of serum biochemical indicators, tissue pathologies and tissue organ indexes, **1i** could effectively reverse the damage to mouse liver and kidney caused by DSS, playing a protective role in liver and kidney of mice. In summary, **1i** was an effective anti-inflammatory reagent and could be developed as a potential drug for anti-UC.

## 1 Introduction

Ulcerative colitis (UC) is a type of inflammatory bowel disease (IBD) characterized by pathological mucosal damage and ulcers. At present, the treatment of UC can achieve clinical relief for most patients, but it is prone to recurrence and has poor long-term efficacy. UC is known as “green cancer”; Patients with UC face a higher risk of developing colon cancer (Eaden et al., 2001; Jess et al., 2012). In addition, most drugs used for its treatment can lead to serious side effects (Li et al., 2023; Harbord et al., 2017; Kawalec and Malinowski, 2015).

The structure-activity relationship (SAR) of drugs refers to the relationship between chemical structures and physiological activities, and is one of the main research topics in medicinal chemistry. Studying the relationship between the chemical structures and biological activities of derivatives can help identify which chemical modifications to the structure can enhance their activity or reduce their toxic effects. Structural analogues dominated by natural active products or known drugs have made significant contributions to the development of drugs.

**FIGURE 1:**
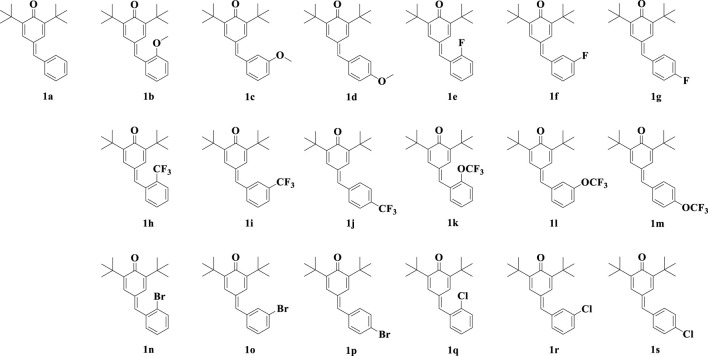
Chemical structures of synthesized *para*-quinone methide derivatives.

Quinone methides are a class of biologically active compounds that can be used as antiviral, antifungal, antibacterial, anti-inflammatory and antioxidant agents in medicine (Kula et al., 2023). In our previous work (Li et al., 2022), we synthesized a series of *para*-quinone methide derivatives with different electron withdrawing/donating groups (methoxy, fluorine, trifluoromethyl, trifluoromethoxy, bromine or chlorine) in the *ortho-*, *meta-*, or *para*-positions of the benzene ring (Figure 1), and systematically studied their SAR. Among them, the derivatives with *ortho*-substitution showed the best anti-proliferation activity in several cancer cells. Especially, the active *ortho*-trifluoromethyl substituted *para*-quinone methide derivative **1h** showed certain safety *in vivo* and could exhibit potential anti-tumor activity by inhibiting thioredoxin reductase (TrxR) *in vivo* and *in vitro*.

Additionally, we found that *ortho*-fluorine, trifluoromethyl, and methoxy substituted hybrid derivatives of curcumin and β-ionone exhibited stronger anti-cancer activity than their corresponding *meta* and *para-*substituted compounds (Yang et al., 2020). While, the hybrid derivatives with *meta*-substitutions exhibited good anti-inflammatory activity in both the inflammatory Raw264.7 cell model induced by lipopolysaccharide (LPS) and the UC mouse model induced by dextran sulfate sodium (DSS) (Ma et al., 2022).

Therefore, in this study, we investigated the anti-inflammatory activity of these synthesized *para*-quinone methide derivatives. Exploring the SAR of nitric oxide (NO) inhibitory activity in the LPS-induced inflammatory Raw264.7 cell model; And investigating the *in vivo* activity of active hybrid derivatives in the DSS-induced UC mouse model.

## 2 Materials and methods

### 2.1 LPS induced NO generation in RAW264.7 cells

Inoculate 100 μL of Raw264.7 cells (SCSP-5036, Center for excellence in molecular cell science of the Chinese academy of sciences) into a 96 well plate at a density of 1 × 10^6^ cells/mL. After 24 h of incubation, discard the original culture medium and add fresh culture medium DMEM (C3113-0500, Hyclone) containing 10% fetal bovine serum (C04001-500, Gibco) and 1% penicillin-streptomycin solution (C0222, Beyotime) containing LPS (1 μg/mL, S1732, Beyotime) and corresponding concentrations of compounds. After continue incubation for 24 h, take 75 μL of culture medium supernatant and 75 μL of freshly prepared Griess reagent (0.1% *N*-(1-naphthyl)-ethylenediamine dihydrochloride, 1% sulfonamide, and 2.5% phosphate) (B010348, B010385 and W810039, Energy Chemical) to a new 96 well plate. Measure the OD value of the mixture solution at 540 nm using a microplate reader (Tecan, Infinite 200 pro). The experiment was performed in triplicate.

### 2.2 Reactive oxygen species (ROS) determination

Inoculate 2 mL of Raw264.7 cells at a density of 1.5 × 10^6^ cells/mL into a 6-well plate. After 24 h of incubation, discard the original culture medium and add fresh culture medium containing LPS (1 μg/mL) and corresponding concentrations of compounds. After incubation for 24 h, collect cells by centrifugation and wash twice with PBS. Add 0.5 mL of DCFH-DA (3 μM) (T88220, Yuanye) solution to each group of cells and incubate at 37°C for 30 min. After washing with PBS three times, the fluorescence intensity (λ_Ex_ = 495 nm and λ_Em_ = 529 nm) of each group of samples was measured using a fluorescence spectrophotometer (Hitachi High-Tech Science, F-7000).

### 2.3 Modeling and drug intervention of UC in mice

After Balb/c mice (8-week-old, 18–20 g, male; Jinan Pengyue experimental animal breeding Co., Ltd.) were adapted for 7 days, they were randomly assigned into five groups (n = 8). Eight normal mice were randomly selected as the normal group. Except for the mice in the normal group, the mice were free to drink 3% DSS (MB5535-2, Meilunbio) aqueous solution for 10 days. For the model group, mice were only free to drink 3% DSS aqueous solution. For the **1i**-treatment groups, starting from the fifth day, mice were orally administered with different concentrations of active derivatives **1i** (50, 100 or 200 mg/kg/day) in carboxymethylcellulose sodium (0.5%) solution (P1346637, Adamas-beta). On the tenth day, after gavage, the mice were fasted for 12 h and were euthanized by cervical dislocation. All operations were strictly carried out in accordance with the National Laboratory Animal Care and Use Guidelines. The animal experiment of this study was approved by the Ethics Committee of Liaocheng University (2022111013).

### 2.4 Determination of serum biochemical indicators

Blood was collected by removing the eyeball and placed in an anticoagulant centrifuge tube. Centrifuge at 4°C and 1,000 g for 10 min, and take the supernatant to obtain serum. Use the aspartate transaminase (AST)/glutamic oxaloacetic transaminase (GOT) activity detection kit (E2023, Applygen), the alanine aminotransferase (ALT)/glutamate pyruvate transaminase (GPT) activity detection kit (E2021, Applygen), the urea (BUN) assay kit (C013-1-1, Nanjing Jiancheng), and the creatinine (CRE) assay kit (C011-2-1, Nanjing Jiancheng) for detection. The content of AST, ALT, BUN and CRE are represented by U/L, U/L, mmol/L and μmol/L, respectively.

### 2.5 Determination of malonic dialdehyde (MDA) content in colon tissue

Mix 50 μL of colon tissue supernatant and 100 μL of working solution (0.4% thiobarbituric acid (w/v) (V900387, Vetec), 0.5% SDS (w/v) (ST627, Beyotime), 9.4% acetic acid (w/v)) (B020052, Energy Chemical). After heating at 100°C for 15 min, the mixture was cooled using the ice bath. After centrifugation, 100 μL of supernatant were placed in a 96 well plate and the absorbance at 532 nm was measured using a microplate reader. The MDA content was expressed in nmol/mg pro.

### 2.6 Determination of myeloperoxidase (MPO) level in colon tissue

Mix 40 μL of colon tissue supernatant and 60 μL of working solution (0.0005% *o*-diaminodiphenylenediamine dihydrochloride (A17175, Alfa Aesar) and PBS (50 mM, pH 6.0) solution of 0.1% hydrogen peroxide). Measure the absorbance change at 460 nm using a microplate reader at 25°C. A change of 5.65 × 10^−3^ in absorbance was equivalent to one unit of MPO activity. MPO activity is expressed in U/mg pro.

### 2.7 Determination of catalase (CAT) level in colon tissue

The CAT level in the colon tissue was detected by the catalase assay kit (A007-2-1, Nanjing Jiancheng). CAT activity was expressed in U/g pro.

### 2.8 Histopathology

Colon tissue was fixed with 4% paraformaldehyde fix solution (P0099, Beyotime), embedded in paraffin, and sliced. Stained with the hematoxylin and eosin (HE) staining kit (C0105S, Beyotime), photographed under an Olympus microscope (BX53 + DP80). Then Colon sections were scored according to the histological scoring system (Wang et al., 2021).

### 2.9 Immunohistochemical analysis

The immunohistochemical (IHC) experiment used conventional methods. The primary antibodies F4/80 (GB113373) was purchased from Servicebio. The slides were stained with DAB (G1212, Servicebio) and counter-stained with hematoxylin (G1004, Servicebio).

### 2.10 Western blotting

#### 2.10.1 Whole cell extraction

Inoculate 2 mL of Raw264.7 cells into a 6-well plate at a density of 1.5 × 10^6^ cells/mL. After 24 h of incubation, discard the original culture medium and add fresh culture medium containing LPS (1 μg/mL) and corresponding concentrations of compounds. After incubation for 24 h, collect cells by centrifugation and wash twice with PBS. Cell lysis buffer for Western and IP (P0013, Beyotime) and protease inhibitor cocktail for general use (P1005, Beyotime) were added to each group of cells for cell lysis. After sufficient lysis, centrifuge at 12,000 g for 5 min, take the supernatant, and measure the protein concentration using the enhanced BCA protein assay kit (P0010, Beyotime). The entire experiment process was conducted under ice bath or 4°C conditions.

#### 2.10.2 Nuclear protein extraction

Inoculate 2 mL of Raw264.7 cells into a 6-well plate at a density of 1.5 × 10^6^ cells/mL. After 24 h of incubation, discard the original culture medium and add fresh culture medium containing LPS (1 μg/mL) and corresponding concentrations of compounds. After incubation for 24 h, collect cells by centrifugation and wash twice with PBS. Each group of cells was used to extract nuclear protein using the nuclear and cytoplasmic protein extraction kit (R0050, Solarbio), and the protein concentration was measured using the enhanced BCA protein assay kit. The entire experiment process was conducted under ice bath or 4°C conditions.

#### 2.10.3 Colon tissue protein extraction

Weigh a certain amount of colon tissue and use the cell lysis buffer for Western and IP and tissue crusher (Shanghai Jingxin) for fragmentation. After sufficient lysis, centrifuge at 12,000 g for 5 min, take the supernatant, and measure the protein concentration using the enhanced BCA protein assay kit. The entire experiment process was conducted under ice bath or 4°C conditions.

#### 2.10.4 The WB experiments used conventional methods

The primary antibodies were as follows: the toll-like receptor 4 (TLR4) (WL00196, Wanleibio), interleukin (IL)-6 (WL02841, Wanleibio), tumor necrosis factor-α (TNF-α) (WL01581, Wanleibio), inducible nitric oxide synthase (iNOS) (WL0992a, Wanleibio), NLR family pyrin domain-containing protein 3 (NLRP3) (WL02635, Wanleibio), Caspase1 (WL03450, Wanleibio), B-cell lymphoma 2-associated X (Bax) (WL01637, Wanleibio), Caspase3 (WL02117, Wanleibio), Ki-67 (WL01384a, Wanleibio), nuclear factor kappa B (NF-κB) p65 (bs-0465R, Bioss), β-actin (81115-1-RR, Proteintech), β-tubulin (WL01931, Wanleibio), Histone H3 (68345-1-IG, Proteintech). The secondary antibodies were as follows: HRP-labeled goat anti-mouse and anti-rabbit IgG (H + L) (A0216 and A0208, Beyotime).

### 2.11 Molecular docking simulation calculation

Simulation calculation of molecular docking between active drug molecule **1i** and myeloid differentiation factor 2 (MD2) (PBD ID, 3VQ2) or cluster of differentiation 14 (CD14) (PBD ID, 4GLP) using the Libdock module of the discovery studio (DS) software. The entire *C*-chain of MD2 (x = −32.237392, y = −17.019290, z = 31.671505; radius = 27.117655) or the *N*-terminal hydraulic pocket of the A-chain of CD14 (x = 48.350000, y = 51.480000, z = 0.970000; radius = 29.370001) was used as the calculation range.

### 2.12 Statistical analysis

Data were expressed using the average and standard deviation values of at least three independent experiments. Statistical significances were analyzed using the Kruskal-Wallis test followed by the Dunns test in the GraphPad Prism software.

## 3 Results

### 3.1 NO production

We measured the inhibitory activity of *para*-quinone methide and eighteen derivatives (Figure 1) modified with methoxy, fluorine, trifluoromethyl, trifluoromethoxy, bromine or chlorine in the *ortho-*, *meta-*, or *para*-positions of the benzene ring on the NO production in the LPS-induced inflammatory Raw264.7 cell model. These synthesized derivatives were prepared and structurally identified according to our previously reported methods, as shown in Supplementary Material.

According to the results in Table 1, for derivatives modified with the same substituent, the *meta*-substituted derivative had the best NO inhibitory activity compared to *ortho-* and *para*-substituted derivatives. For *meta*-substituted derivatives, *meta*-trifluoromethyl modified derivative had the highest anti-inflammatory activity, which was selected for subsequent research. In addition, we measured the cytotoxicity of the derivatives on Raw264.7 cells (Supplementary Table S1). Some derivatives had certain cytotoxic activity. However, the cytotoxic activity of most compounds didn’t affect their ability to inhibit NO production, including **1i** (Supplementary Figure S1).

**TABLE 1:** The inhibitory activity of *para*-quinone methide derivatives on the NO production.

Coms	IC_50_/μm	Coms	IC_50_/μm	Coms	IC_50_/μm
1b	26.63 ± 2.61	1h	10.20 ± 0.96	1n	23.78 ± 0.60
1c	13.37 ± 1.15	1i	3.87 ± 0.35	1o	8.17 ± 0.78
1d	18.30 ± 1.82	1j	7.97 ± 0.61	1p	12.53 ± 0.91
1e	15.83 ± 1.19	1k	7.17 ± 0.64	1q	6.57 ± 0.42
1f	9.00 ± 0.53	1l	5.70 ± 0.44	1r	6.07 ± 0.58
1g	10.13 ± 0.93	1m	11.23 ± 0.47	1s	11.50 ± 0.40
1a	12.80 ± 1.20				

### 3.2 ROS production

**FIGURE 2:**
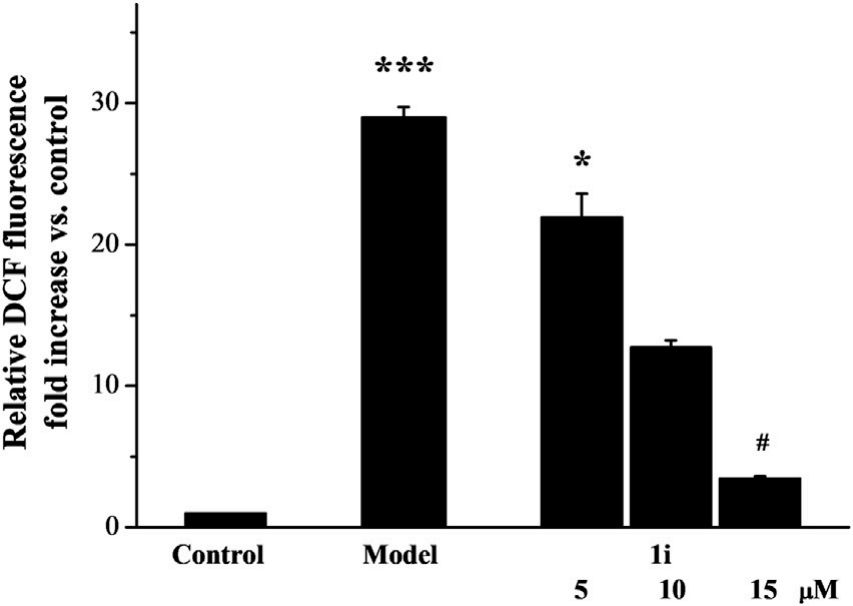
The effect of active derivative **1i** on LPS-induced excess production of ROS. *p < 0.05 and ***p < 0.001, compared with the control group. ^#^ *p* < 0.05, compared with the model group. The experiment was performed in triplicate.

As shown in Figure 2, LPS induced excessive accumulation of ROS in Raw264.7 macrophage cells. The intervention of the active derivative **1i** can reduce the production of ROS in a dose-dependent manner.

### 3.3 Expressions of inflammatory related proteins

**FIGURE 3:**
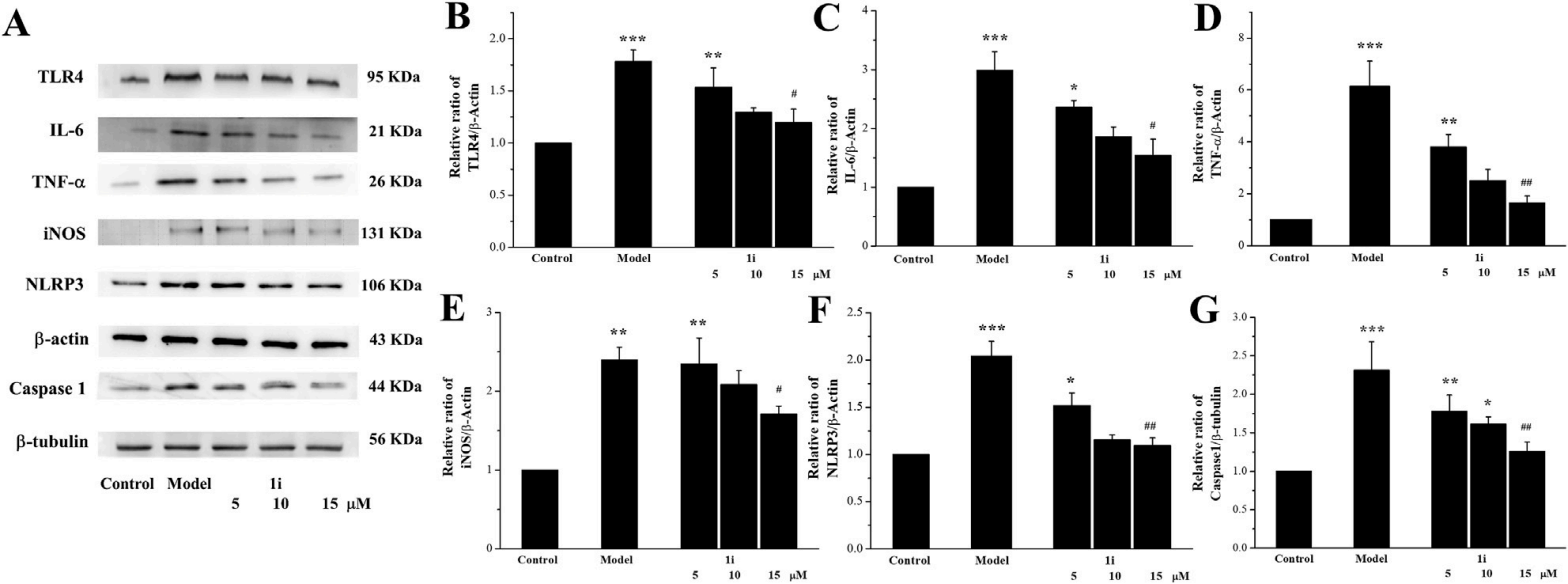
Effects of the active derivative **1i** on expressions of inflammatory related proteins in LPS-induced Raw264.7 cells. **(A)** Representative protein bands. Quantitative analysis of **(B)** TLR4, **(C)** IL-6, **(D)** TNF-α, **(E)** iNOS, **(F)** NLRP3 and **(G)** Caspase 1 grayscale using the ImageJ software. *p < 0.05, **p < 0.01 and ***p < 0.001, compared with the control group. ^#^ *p* < 0.05 and ^##^ *p* < 0.01, compared with the model group. The experiment was performed in triplicate.

We evaluated the effects of **1i** on the expressions of inflammation related proteins TLR4, IL-6, TNF-α, NLRP3, and Caspase 1. In addition, we also measured the expression of inducible iNOS, a regulatory factor for inflammatory NO synthesis. As shown in Figure 3, LPS induced overexpressions of TLR4, IL-6, TNF-α, iNOS, NLRP3, and Caspase 1 in Raw264.7 macrophage cells. And the intervention of **1i** resulted in a decrease in these overexpressions in a dose-dependent manner.

**FIGURE 4:**
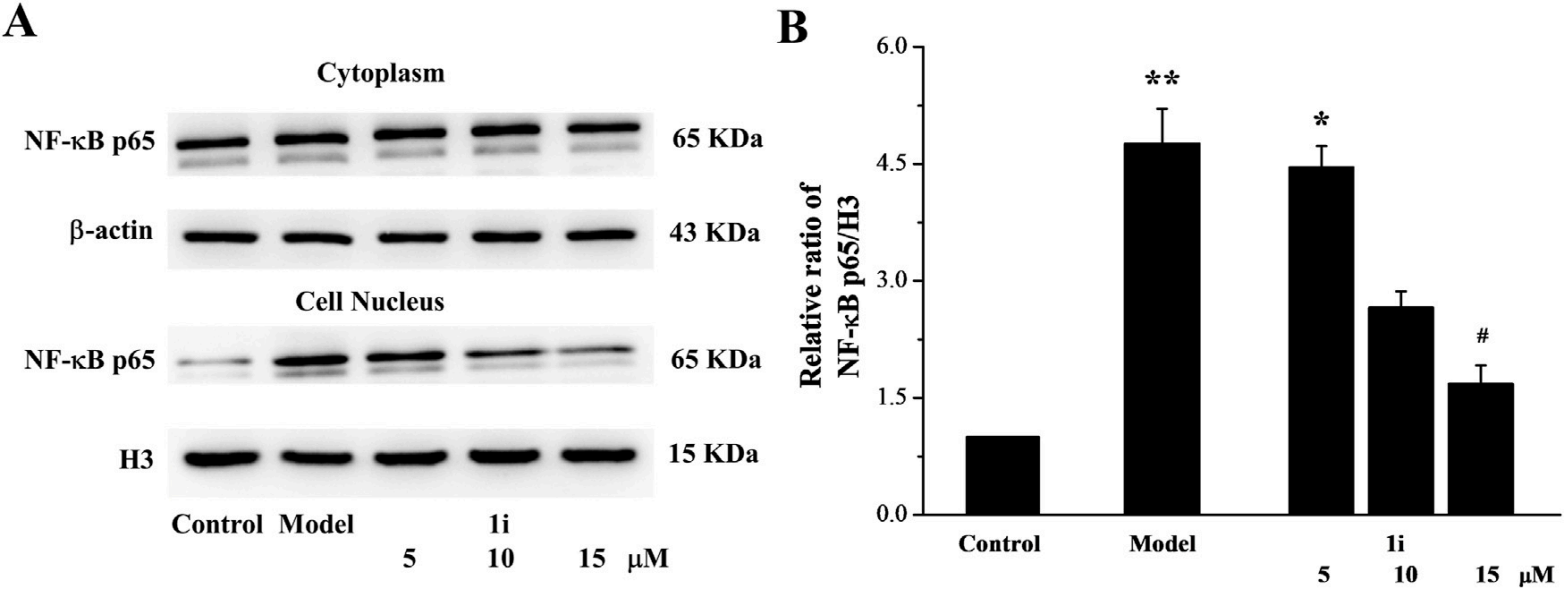
The effect of **1i** on nuclear transfer of NF-κB protein. **(A)** Representative protein bands. **(B)** Perform grayscale quantitative analysis of NF-κB in nuclear proteins using the ImageJ software. *p < 0.05 and **p < 0.01, compared with the control group. ^#^ *p* < 0.05, compared with the model group. The experiment was performed in triplicate.

The nuclear transfer of NF-κB protein expression was also determined. As shown in Figure 4, LPS induced the increase in NF-κB expression in the nucleus. And the intervention of **1i** reduced the nuclear transfer of NF-κB.

### 3.4 Molecular simulation calculations

TLR4-NF-κB is a key pathway for initiating inflammatory responses. TLR4 is a key receptor or promoter for transmembrane signal transduction of LPS. The recognition of TLR4 and LPS involves the binding of LPS to the CD14 receptor, and the transmitting LPS to the TLR4-MD2 complex. TLR4 recognizes LPS with the help of CD14 and MD2. In order to better understand the intervention effect of **1i** on the TLR4-NF-κB signaling pathway, we used the Libdock module of DS to perform molecular docking simulation calculations between **1i** and CD14 or MD2. The *N*-terminal hydrophobic pocket of the A-chain of CD14 and the C-chain of the MD2 were defined as binding sites for calculation.

**FIGURE 5:**
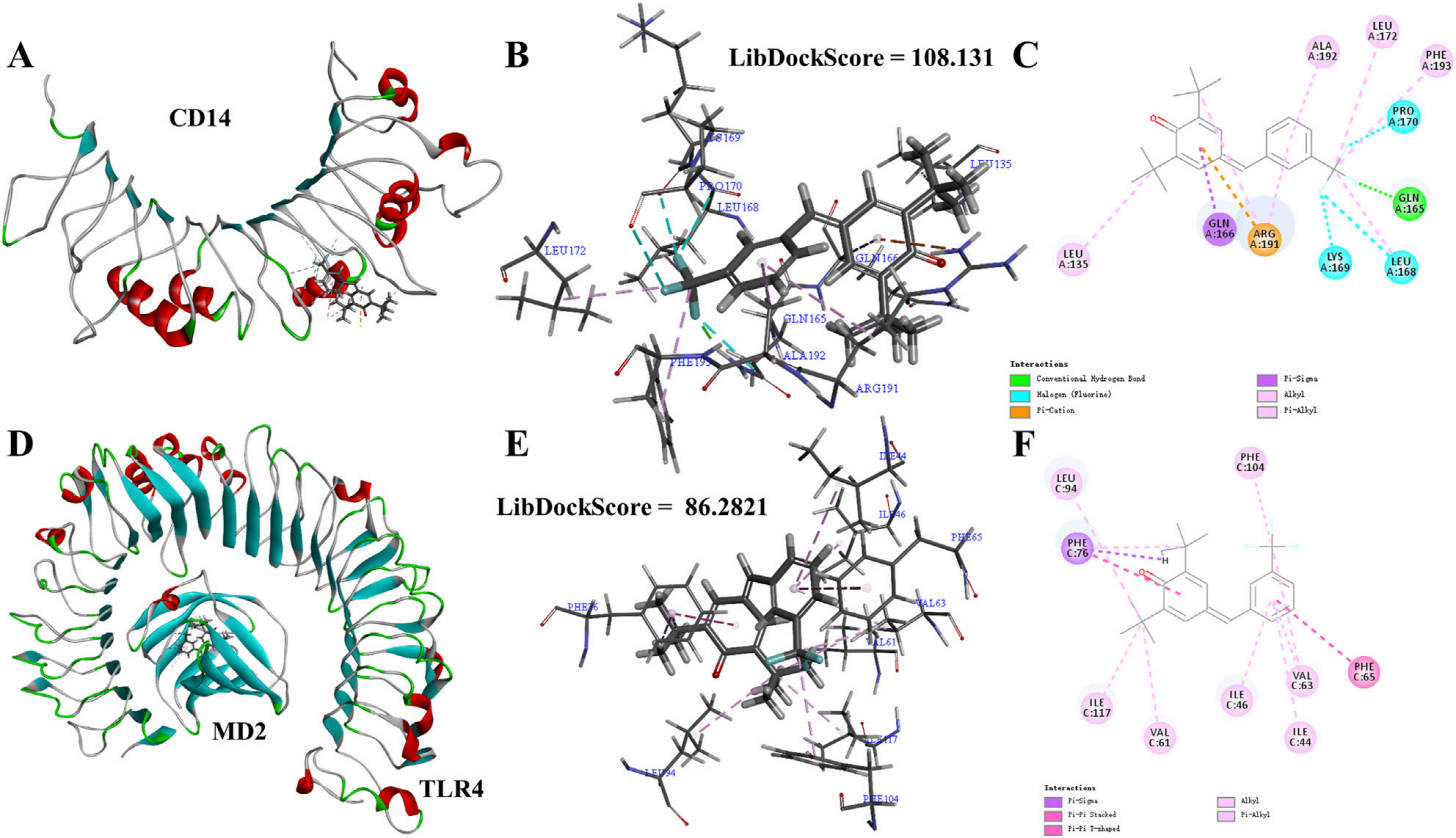
Molecular simulation calculations of **1i** and CD14 and TLR4-MD2. **(A–C) 1i**-CD14 interactions on 3D and 2D diagrams. **(D–F) 1i**-MD2 interactions on 3D and 2D diagrams.

As shown in Figure 5, **1i** can form molecular docking with both CD14 and MD2. **1i** formed the conventional hydrogen bond, halogen, pi-cation, pi-sigma, alkyl, and pi-alkyl with CD14. While, **1i** formed pi-sigma, pi-stacked, pi-T-shaped, alkyl, and pi-alkyl with MD2. There were more interactions between **1i** and CD14 than MD2, and there was the stable conventional hydrogen bond. Additionally, the Libdockscore for molecular docking between **1i** and CD14 was 108.131, higher than the Libdockscore of 86.2821 for molecular docking between **1i** and MD2. These results suggested that the inhibition of CD14 by **1i** may have a more effective anti-inflammatory effect.

### 3.5 *In vivo* study

#### 3.5.1 Effects of symptoms on the mouse model of UC

**FIGURE 6:**
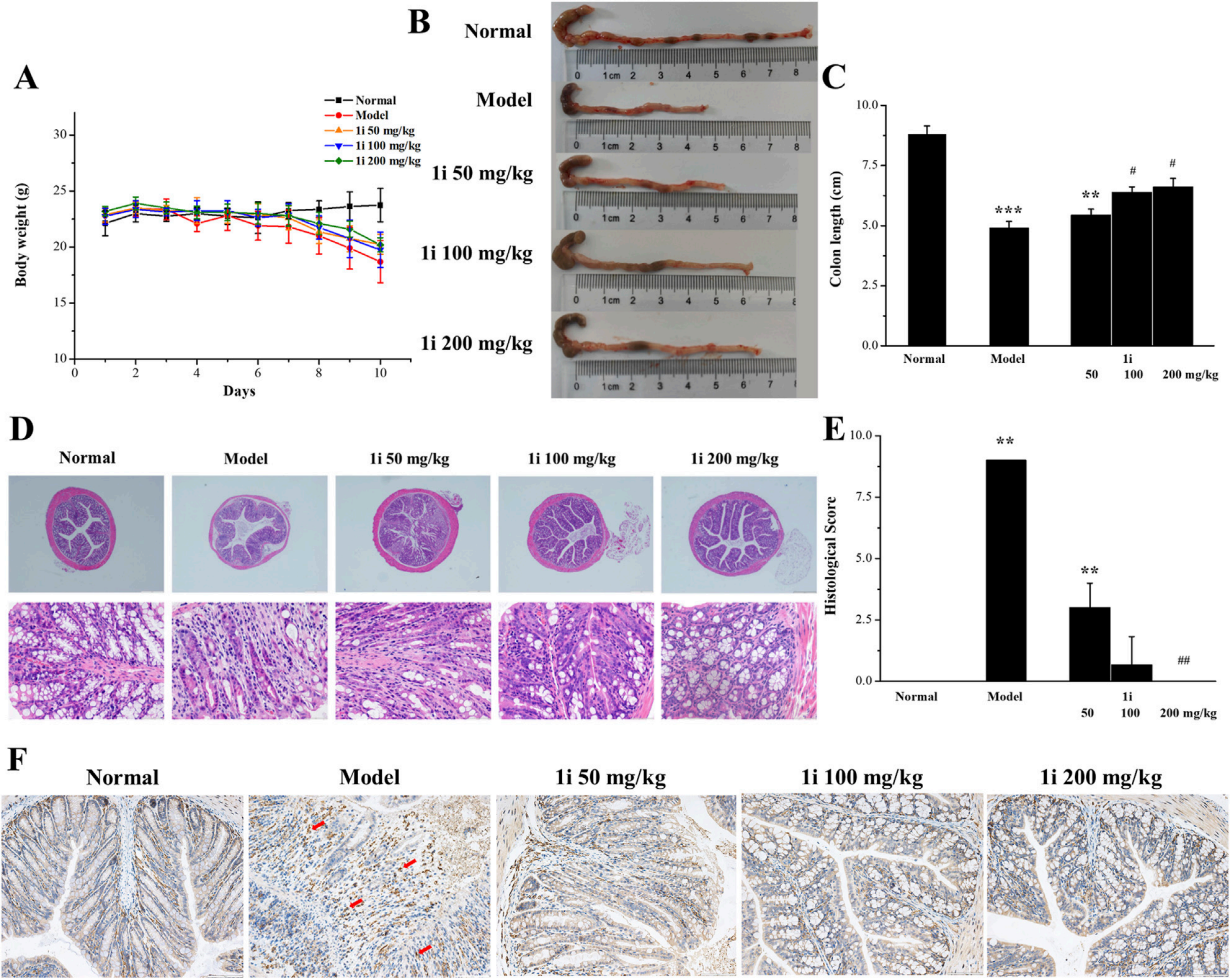
The anti-inflammatory activity of **1i** in mice with UC. **(A)** Changes in mouse body weight; **(B, C)** colon length; **(D)** Representative images of histopathological analysis of colon tissue stained with HE. **(E)** Colon sections were scored according to the histological scoring system. **(F)** Representative images of histopathological analysis of colon tissue stained with F4/80. **p < 0.01 and ***p < 0.001, compared with the normal group. ^#^ *p* < 0.05 and ^##^ *p* < 0.01, compared with the model group. Scale bar: D, 500 μm and 50 μm; F, 100 μm. The number of mice per group was 8.

Further study on the anti-inflammatory activity of **1i** *in vivo* using DSS-induced UC mouse model. The experimental mice were free to drink 3% DSS solution for 10 days; And starting from the fifth day, the experimental mice were treated with the active derivative **1i** by gavage for 6 days. As shown in Figure 6A, as the drinking time of DSS increased, the weight of mice decreased in the model group. In the drug treatment group, the weight loss of mice slightly decreased after gavage with **1i**. For the length of the colon, as shown in Figures 6B, C, DSS significantly shortened the length of the colon; After gavage, the length of the colon increased in a dose-dependent manner. For the pathological tissue sections of the colon, as shown in Figures 6D, E, compared with the normal group, the pathological tissue sections of the colon in the model group showed obvious inflammatory reactions, such as disappearance of crypts, inflammatory infiltration and structural destruction. When treated with **1i** by gavage, a dose of 50 mg/kg of **1i** can alleviate these inflammatory reactions; 100 mg/kg of **1i** significantly improved these inflammatory responses; 200 mg/kg of **1i** can basically reverse these inflammatory reactions. IHC staining of colon tissue with F4/80, a recognized biomarker for mouse macrophages, showed that DSS induced massive macrophage infiltration in the colon of UC mice; While, the intervention of **1i** significantly inhibited macrophage infiltration, as shown in Figure 6F. These results suggested that **1i** can protect colon tissue from damage caused by DSS.

#### 3.5.2 Effects of oxidative stress on the mouse model of UC

**FIGURE 7:**
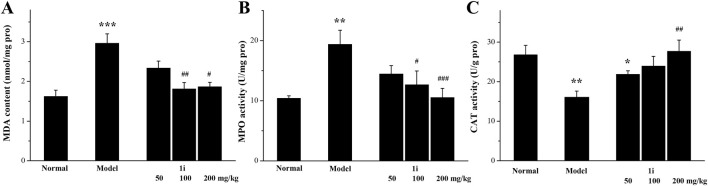
Determination of oxidative stress indicators in colon tissue. **(A)** MDA, **(B)** MPO and **(C)** CAT. *p < 0.05, **p < 0.01 and ***p < 0.001, compared with the normal group. ^#^ *p* < 0.05, ^##^ *p* < 0.01 and ^###^ *p* < 0.001, compared with the model group. The number of mice per group was 8. The experiment was performed in triplicate.

As shown in Figure 7, in the model group, the MDA and MPO levels in the colon tissue of UC mice were significantly increased; However, the expressions of CAT were significantly reduced. This meant that the oxidative stress level in the colon tissue of UC mice was increased. Oral administration of the active derivative **1i** to UC mice can reverse these changes in a dose-dependent manner.

#### 3.5.3 Regulation of inflammatory related proteins in colon tissue

**FIGURE 8:**
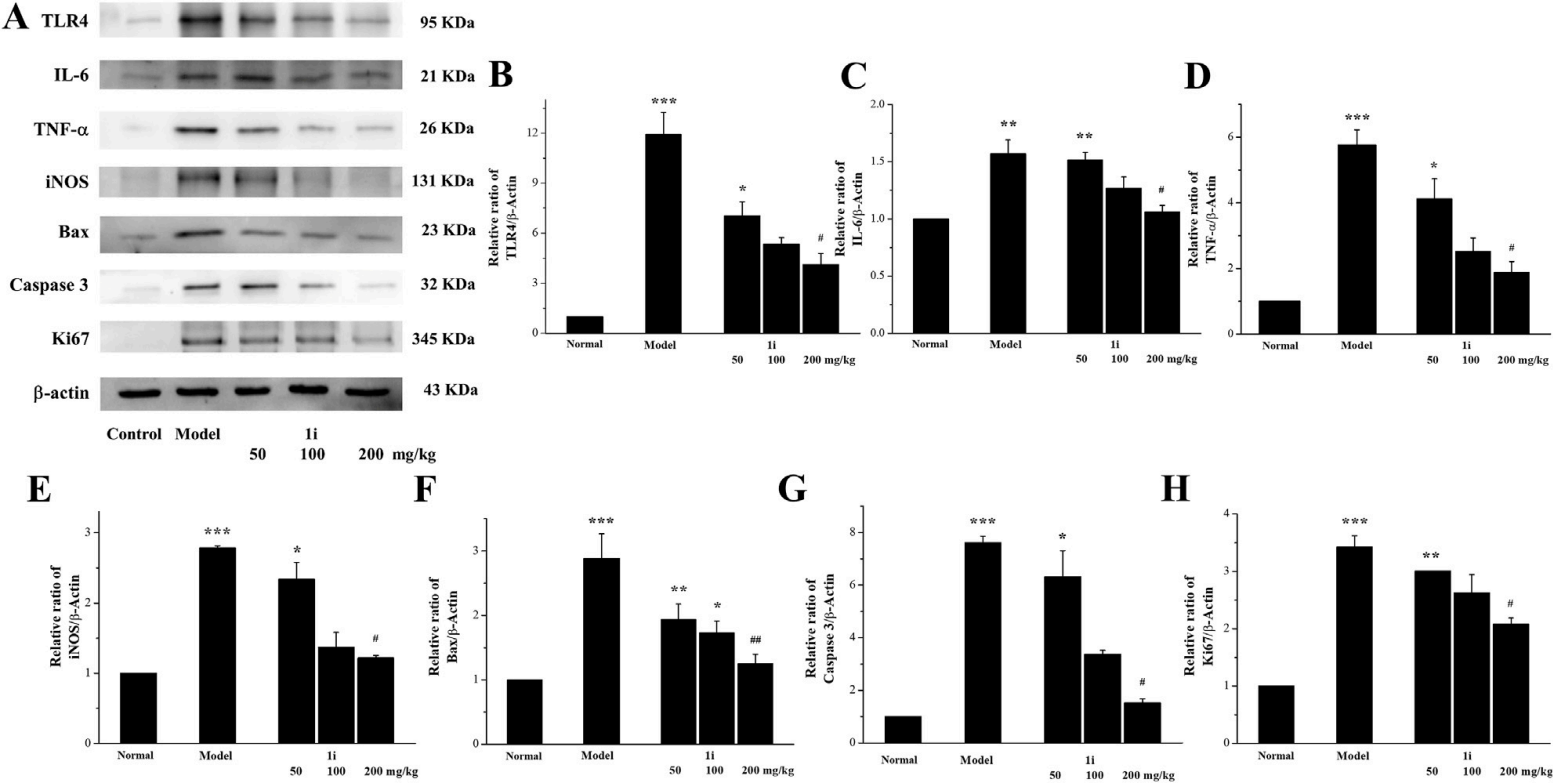
Effects of the active derivative **1i** on expressions of inflammatory related proteins in DSS-induced UC mice. **(A)** Representative protein bands. Quantitative analysis of **(B)** TLR4, **(C)** IL-6, **(D)** TNF-α, **(E)** iNOS, **(F)** Bax, **(G)** Caspase 3 and **(H)** Ki67 grayscale using the ImageJ software. *p < 0.05, **p < 0.01 and ***p < 0.001, compared with the normal group. ^##^ *p* < 0.01 and ^###^ *p* < 0.001, compared with the model group. The experiment was performed in triplicate.

We measured the expressions of inflammation related proteins TLR4, IL-6, TNF-α, and iNOS in colon tissue. As shown in Figures 8A–E, compared with normal mice, the expressions of TLR4, IL-6, TNF-α, and iNOS in UC mice were significantly increased, while the active derivative **1i** could reduce these expressions of these inflammation related proteins.

#### 3.5.4 Regulation of apoptosis related proteins in colon tissue

We further detected the expression of two pro-apoptotic proteins, Bax and Caspase 3, to confirm whether the active derivative **1i** can regulate the expression of apoptosis related proteins to resist damage caused by UC. As shown in Figures 8A, F, G, the expressions of pro-apoptotic proteins Bax and Caspase 3 in the colon tissue of UC mice were indeed increased, consistent with literature reports. The intervention of the active derivative **1i** can effectively reduce the expressions of these two pro-apoptotic proteins. The effect of **1i** on cell apoptosis has also been confirmed by the cell system, that **1i** can reverse LPS-induced apoptosis in Raw264.7 cells (Supplementary Figure S2).

#### 3.5.5 Regulation of Ki67 in colon tissue

In addition, we measured the expression of Ki67 in mouse colon tissue. As shown in Figures 8A, H, compared with the normal group, the colon tissue of UC mice showed high expression of Ki67; However, the expression of Ki67 was decreased with increasing **1i** concentration. This result indicated that **1i** can reduce DSS-induced colon tissue damage in UC mice by inhibiting the expression of Ki67.

#### 3.5.6 Evaluation of liver and kidney function

**FIGURE 9:**
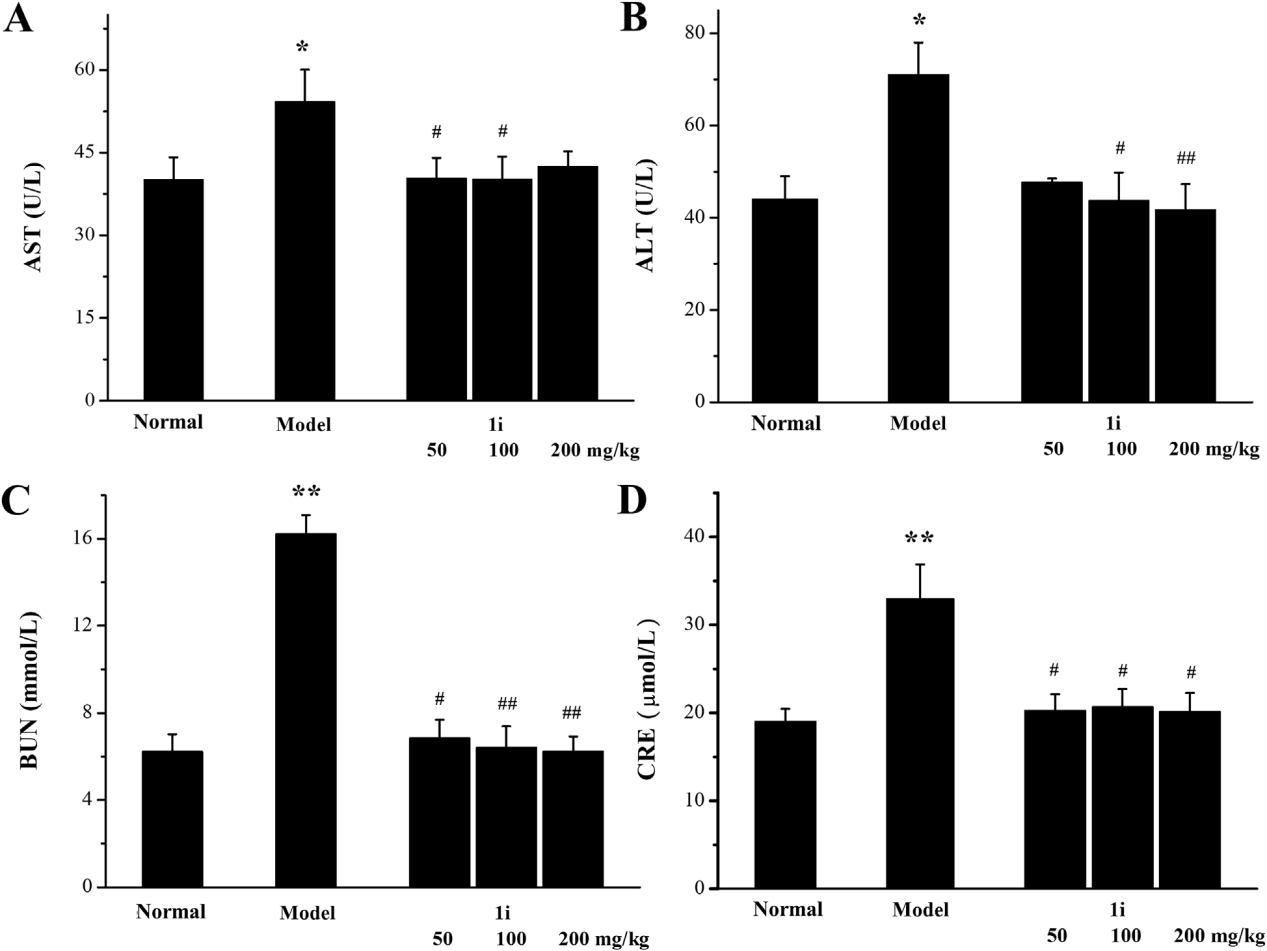
Evaluation of biochemical indicators of liver and kidney function in serum. **(A)** AST level. **(B)** ALT level. **(C)** BUN level. **(D)** CRE level. *p < 0.05, **p < 0.01, compared with the normal group. ^#^ *p* < 0.05 and ^##^ *p* < 0.01, compared with the model group. The number of mice per group was 8. The experiment was performed in triplicate.

We further used serum biochemical indicators (AST, ALT, BUN and CRE), pathological tissue analyses, and the organ indexes to evaluate the liver and kidney function damage. As shown in Figure 9, in the UC model group, the levels of AST, ALT, BUN, and CRE in the serum of the model mice were higher than those of normal mice, indicating that DSS caused damage to liver and kidney function in the UC model mice. The treatment of the active derivative **1i** resulted in the decreases in AST, ALT, BUN and CRE levels, indicating that the intervention of **1i** can effectively reverse the liver and kidney function damages caused by DSS.

**FIGURE 10:**
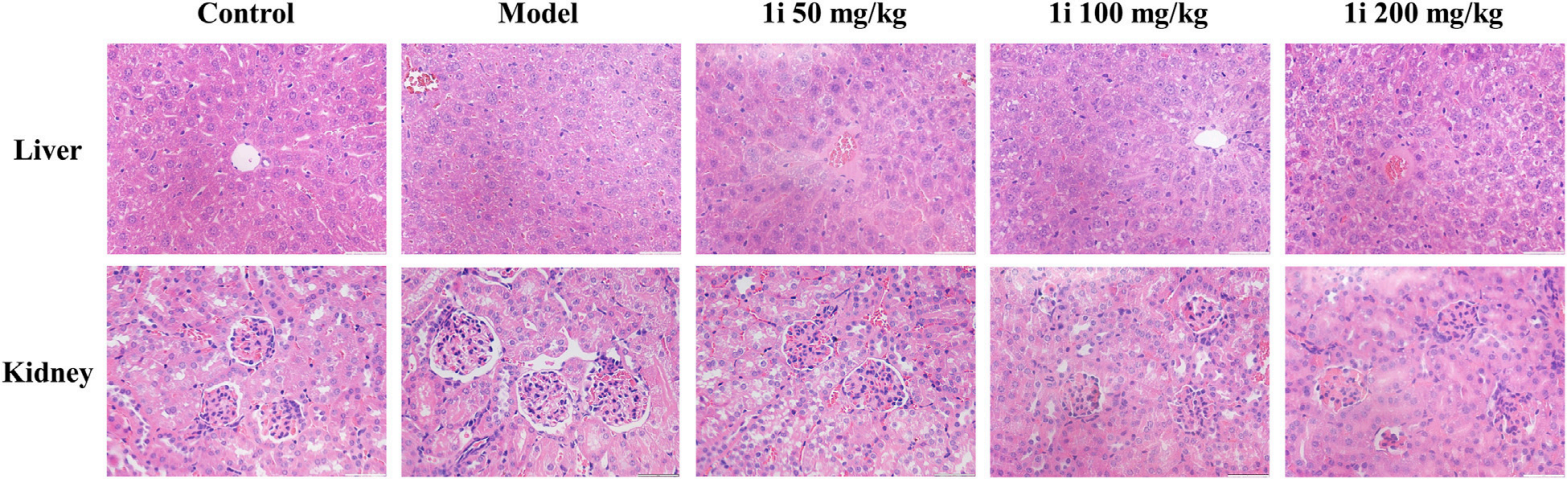
Representative images of pathological tissue sections of liver and kidney. Scale bar: 50 and 50 μm. The number of mice per group was 8.

However, as shown in Figure 10, there was no significant change in the pathological examination of liver tissue between the groups; As shown in Table 2, there was no statistically significant difference in the liver organ index between the groups.

**TABLE 2:** Organ indexes of liver and kidney of DSS-induced mice.

Groups	Liver	Kidney
Control	0.039 ± 0.0036	0.014 ± 0.0011
Model	0.039 ± 0.0039	0.017 ± 0.0012*
**1i** 50 mg/kg	0.041 ± 0.0040	0.015 ± 0.0012^#^
**1i** 100 mg/kg	0.041 ± 0.0031	0.015 ± 0.0003^#^
**1i** 200 mg/kg	0.040 ± 0.0038	0.014 ± 0.0012^#^

*p < 0.05, compared with the normal group; ^#^ *p* < 0.05, compared with the model group. The number of mice per group was 8. The experiment was performed in triplicate.

For the kidneys, as shown in Figure 10, pathological examination revealed an increase in glomerular volume in the UC model group; As shown in Table 2, the organ index of the kidney in the UC model group significantly increased. And the intervention of **1i** can reverse these changes.

## 4 Discussion

The research on natural products has shown great potential in the field of new drug development. Quinones are natural organic compounds that are widely present in nature, and possess various biological activities. Numerous studies have shown that quinones and their derivatives have clear anti-inflammatory effects, and their development potential as anti-inflammatory drugs are enormous (Guo et al., 2024). Quinone methides are the derivatives of quinones, in which one oxygen atom is replaced by carbon; Quinone methides have stronger polarity and higher reactivity. Most quinone methides exhibit significant biological activity. For example, celastrol, a quinone methide triterpenoid, can inhibit the inflammatory response of macrophages and resist the invasion of skin inflammation, enteritis, arthritis, Alzheimer’s disease, and so on (Zhao et al., 2015; Kim et al., 2009; Allison et al., 2001).

In this study, a series of synthesized *para*-quinone methide derivatives were investigated their anti-inflammatory activity in the LPS-induced inflammatory Raw264.7 cell model. The LPS-induced inflammatory Raw264.7 cell model is a commonly used cell model *in vitro* for anti-inflammatory activity research. LPS can induce excessive production of NO in Raw264.7 cells. NO is an important regulatory molecule in the inflammatory response (Wang et al., 2013). Through the screening of LPS-induced inflammatory cell models in Raw264.7 cells, it was found that the inhibitory activity of *meta*-substituted derivatives on NO production was superior to that of *ortho*- and *para*-modified derivatives. Among them, in the inflammatory cell model, the *meta*-trifluoromethyl substituted *para*-quinone methide derivative **1i** had the best activity in the inhibiting LPS-induced excess generation of NO.

In our previous work (Li et al., 2022), derivatives with *ortho*-modification showed the best anti-proliferation activity against cancer cells. This was consistent with the SAR of hybrid derivatives of curcumin and β-ionone discovered in previous work (Yang et al., 2020; Ma et al., 2022), where *ortho*-substituted derivatives exhibited good anti-cancer activity, while *meta*-substituted derivatives exhibited good anti-inflammatory activity.

Inflammation can cause oxidative stress, which can also cause inflammation. The excessive production of ROS and cellular redox imbalance play a core role in the pathophysiology of inflammatory response (Lee and Yang, 2012). A large amount of evidence suggests that oxidative stress is an important pathogenic factor in the inflammatory response (Barrett et al., 2017). In the LPS-induced Raw264.7 macrophage cells, excessive production of ROS is closely related to pathological states such as inflammation. In the LPS-induced Raw264.7 macrophage cells, **1i** can effectively reduce the excessive accumulation of ROS induced by LPS, which means that **1i** can reduce the degree of oxidative stress of the cellular redox imbalance.

In the LPS-induced inflammatory Raw264.7 cell model, LPS first recognizes and forms complexes with the lipopolysaccharide binding protein in the cytoplasm. Further deliver LPS to the leukocyte differentiation antigen CD14 on the cell membrane and decompose complexes into monomeric LPS, which is then presented to the TLR4-MD2. The binding of TLR4-MD2 complex to LPS induces activation of NF-κB, which is a key nuclear factor in the initiation and regulation of inflammatory responses. Activated NF-κB undergoes nuclear transfer, further promoting transcription and translation of cytokines (such as IL-6, IL-1β and TNF-α) and NLRP3. After NLRP3 inflammasome is activated, it further activates Caspase 1, which is mainly involved in the activation of pro-inflammatory cytokines and the process of cell apoptosis.

**1i** can effectively inhibit the expressions of inflammation related initiating protein TLR4, pro-inflammatory cytokines IL-6 and TNF-α, inflammasome NLRP3 and Caspase1, and can reduce the nuclear transfer of NF-κB. These results suggested that **1i** can resist LPS-induced inflammation in Raw264.7 macrophage cells. These results suggested that **1i** can resist LPS-induced inflammation in Raw264.7 macrophage cells.

The DSS-induced UC mouse model is selected for the *in vivo* anti-inflammatory activity evaluation of **1i**. The DSS-induced UC mouse model can simulate the clinical characteristics of human UC, including weight loss, diarrhea, hematochezia, colon shortening, intestinal mucosal damage, and elevated inflammatory markers.

In the DSS-induced UC mouse model, the active derivative **1i** can inhibit DSS-induced colon shortening, and reverse DSS-induced pathological changes in colon tissue, such as inflammatory infiltration, structural destruction, and crypt disappearance. In addition, like the LPS-induced inflammatory Raw264.7 cell model *in vitro*, **1i** can effectively inhibit inflammation related proteins TLR4, IL-6, TNF-α and iNOS in the colon tissue of the DSS-induced UC mouse model. These indicates that **1i** can effectively resist DSS-induced colon inflammation in UC mice.

The pathogenesis of UC is related to increased inflammation and weaken antioxidant capacity. Oxidative stress metabolites are potential inflammatory mediators of IBD (Sheethal et al., 2020). MDA, MPO, and CAT were measured to evaluate the oxidative stress levels in colon tissue. MDA is the final product of lipid peroxidation and an indirect indicator of oxidative stress (Jentzsch et al., 1996). MPO is a peroxidase present in the phenylalanine blue granules of myeloid cells (neutrophils and monocytes) (Palla et al., 2016). Under physiological conditions, MPO is a part of the natural immune system; While, under specific conditions, MPO can catalyze the production of excess oxidants leading to oxidative stress (Bo et al., 2017). CAT protects cells from oxidative stress by catalyzing the breakdown of hydrogen peroxide into water and oxygen (Sepasi Tehrani and Moosavi-Movahedi, 2018). The active derivative **1i** can effectively reverse the increases of MDA and MPO caused by oxidative stress, as well as the decrease of CAT. These indicates that **1i** can effectively resist oxidative stress in UC mice, thereby exhibiting anti-UC effects.

According to literature reports, UC is associated with apoptosis. Excessive apoptosis of colonic epithelial tissue can lead to structural damage and intestinal mucosal barrier damage, thereby inducing inflammation of the colon (Wei et al., 2011; Liu et al., 2021; Cai et al., 2022; Li et al., 2020). The Bcl-2/Bax cell signaling pathway and its downstream caspase family are important proteins related to cell apoptosis. **1i** can reduce the increased expressions of pro-apoptotic proteins Bax and Caspase 3 in the colon tissue of UC mice, which indicates that **1i** can resist UC damage by inhibiting colon apoptosis in UC mice. It is interesting that in our previous work (Li et al., 2022), *meta*-trifluoromethyl modified derivative **1h**, a structural analogue of **1i**, can exert anti-cancer effects by promoting cancer cell apoptosis via targeting TrxR.

UC is one of the triggering factors for colon cancer, and UC patients face a higher risk of developing colon cancer. Ki67 is a proliferating cell related antigen and a commonly used tumor proliferation index in clinical practice. The expression level of Ki67 usually indicates the activity level of cell proliferation (Ilyas and Talbot, 1995). In clinical practice, the higher the expression of Ki67, the more active the proliferation of cells and the higher the degree of malignancy. **1i** can decrease the high expression of Ki67 caused by UC, thereby reducing the colon tissue damage caused by UC.

In the UC mouse model induced by DSS, in addition to causing UC, DSS can also accumulate and cause damage in other organs of the mouse (Yang and Merlin, 2024). AST and ALT are serum biochemical indicators used to evaluate liver damage, while BUN and CRE are serum biochemical indicators used to evaluate kidney damage. Through serum biochemical index measurement, it is found that **1i** can reduce the elevation of AST, ALT, BUN, and CRE caused by UC, thereby reversing DSS-induced liver and kidney damage. Both the histopathological examination and organ index of the kidney tissue show that **1i** can effectively reverse the kidney damage caused by DSS. These results indicate that **1i** can effectively protect against liver and kidney damages caused by DSS, and also confirms the safety of **1i** as the potential anti-UC reagent.

In summary, **1i** is an effective anti-inflammatory reagent and could be developed as a potential drug for anti-UC. Further research will focus on the evaluation of the activity of the active derivative **1i** in other inflammatory models, more detailed mechanisms of action, pharmacokinetics, bioavailability, and evaluations of acute and chronic toxicity.

In addition, the SAR between the anti-cancer and anti-inflammatory properties of this series of *para*-quinone methides derivatives shows that *ortho*-substituted derivatives have good anti-cancer activity, while, *meta*-substitued derivatives have good anti-inflammatory activity. This type of SAR had also been observed in previous studies of hybrid derivatives of curcumin and β-ionone (Yang et al., 2020; Ma et al., 2022). This provides some guidances for the design of drugs containing Michael receptor structures attached to the benzene ring. Further research is needed on computer simulation calculations related to this SAR.

## Data Availability

The original contributions presented in the study are included in the article/Supplementary Material, further inquiries can be directed to the corresponding authors.
