# Supplementary material for: Anti-inflammatory effects of para-quinone methide derivatives on ulcerative colitis
Source: Front Pharmacol. 2024 Oct 29;15:1474678. doi: 10.3389/fphar.2024.1474678 (PMC11554457; doi:10.3389/fphar.2024.1474678)
Supplement: Supplementary file 1 [file DataSheet1.pdf]

Anti-inflammatory effects of *para*-quinone methide derivatives on ulcerative colitis

Yue Qiu[a], Xin Li[a], Xu Zhang[a], Xiaotong Wang[a], Xuekun Wang[a,*], Jie Yang[a,b,*], and Guoyun Liu[a,b,*]

[a]*State Key Laboratory for Macromolecule Drugs and Large-scale Manufacturing, School of Pharmaceutical Sciences, Liaocheng University, Liaocheng 252059, China*

[b]*Liaocheng Key Laboratory of Quality Control and Pharmacodynamic Evaluation of Ganoderma lucidum, Liaocheng University, 1 Hunan Street, Liaocheng, Shandong 252059, China*

Supporting Information

* Corresponding author

Fax: (+86) 15063505132

E-mail: guoyunliu@126.com; yangjie1110@163.com; xuekunwang0610@126.com.

1. Cytotoxicity of *para*-quinone methide derivatives against Raw264.7 cells

Table S1. Cytotoxicity of *para*-quinone methide derivatives against Raw264.7 cells.

Coms	IC_{50}/μM	Coms	IC_{50}/μM	Coms	IC_{50}/μM
**1b**	>100	**1h**	43.44 ± 3.86	**1n**	55.91 ± 0.88
**1c**	>100	**1i**	36.34 ± 2.28	**1o**	34.04 ± 0.27
**1d**	>100	**1j**	38.00 ± 3.93	**1p**	>100
**1e**	64.59 ± 3.80	**1k**	30.95 ± 1.44	**1q**	>100
**1f**	>100	**1l**	31.93 ± 0.33	**1r**	69.65 ± 2.29
**1g**	>100	**1m**	23.47 ± 0.02	**1s**	>100
**1a**	>100				

Inoculate 100 μL of Raw264.7 cells into a 96-well plate at a density of 1 x 10^6 cells/mL. After 24 hours of incubation, discard the original culture medium and add fresh culture medium DMEM containing 10% fetal bovine serum and 1% penicillin-streptomycin solution containing corresponding concentrations of compounds. After continue incubation for 24 hours, add MTT solution and incubate for 4 hours. After removing the medium from the 96-well plate, add 200 μL DMSO to dissolve the formazan. Take 100 μL DMSO to a new 96-well plate, and measure the OD value of the solution at 570 nm. The experiment was performed in triplicate.

2. Effect of 1i on cell viability in Raw264.7 cells, and NO production in LPS-induced Raw264.7 cells.

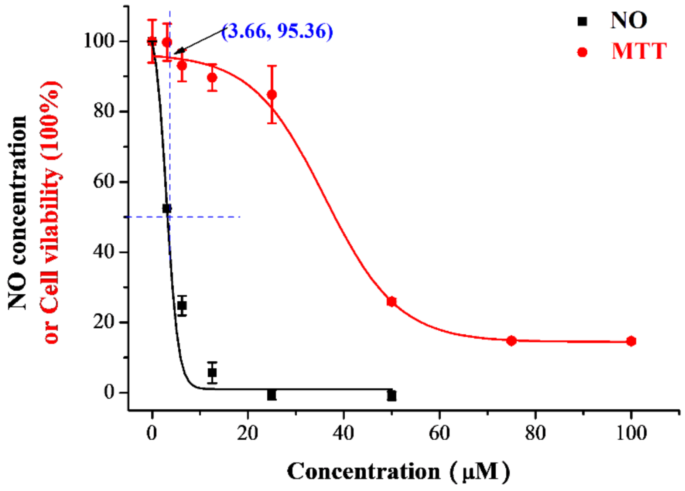

Figure S1. Effects of **1i** on cell viability in Raw264.7 cells, and on NO production in LPS-induced Raw264.7 cells.

3. The effect of 1i on the cell apoptosis in LPS-induced Raw264.7 cells.

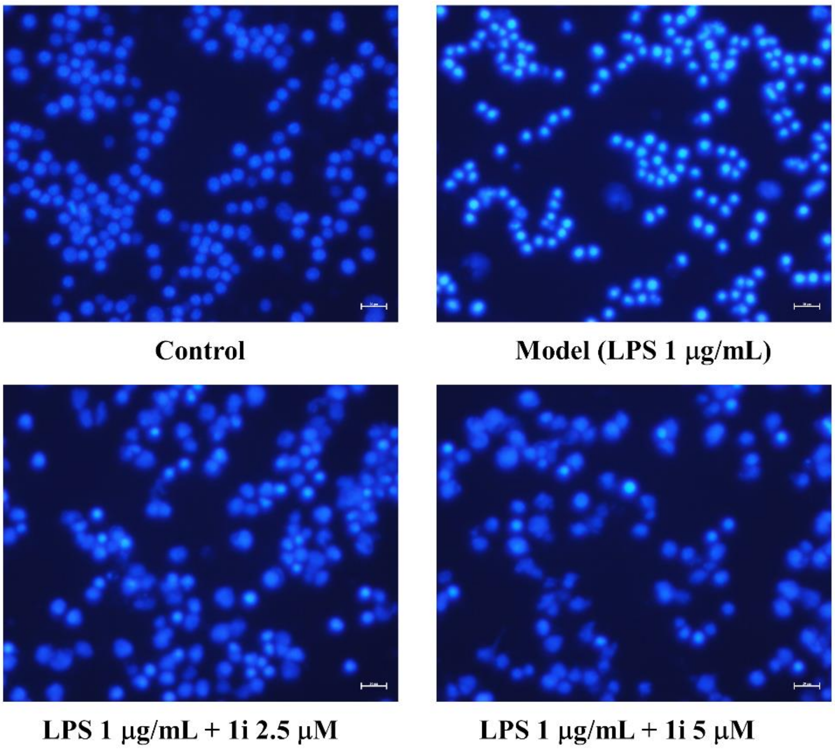

Figure S2. The effect of **1i** on the cell apoptosis in LPS-induced Raw264.7 cells. Scale bar: 20 μm

2 mL of Raw264.7 cells were seeded at a density of 1.5 × 10^5 cells/mL in the six-well plate, and were incubated for 24 h. LPS (1 μg/mL) and different concentrations of **1i** (5 and 10 μM) were added to treat cells for 24 h. After removing the medium, 1 mL of 4% paraformaldehyde fix solution was added to each well to fix the cells for 10 min. After washing the cells twice, 1 mL of Hoechst-33258 (10 μg/mL) was added to stain cells at 37 °C for 15 min in the dark. After the cells were washed with PBS three times, a drop of anti-quenching sealing solution was dropped to the six-well plate, and then the glass slide was covered. The cells were observed and taken photos using a fluorescence microscope.

4. Synthesis and structural characteristics of derivatives

The *para*-quinone methide derivatives were synthesized according to the published procedure[1]. 2,6-di-*tert*-butylphenol (5 mmol) and the corresponding aldehydes (5 mmol) were dissolved in toluene (20 mL) and heated to 100 °C. Piperidine (10 mmol) was dropwise added within 1 h and the reaction mixture was continued to reflux for 3-4 h. After cooling to 100 °C, acetic anhydride (10 mmol) was added to the mixture and stirring for 15 min. Then, the mixture was cooling to room temperature and poured into water. After extracting with CH_2Cl_2, the organic phases were removed under reduced

pressure, and the residue was purified by a silica gel column.

2,6-di-*tert*-butylcyclohexa-4-benzylidene-2,5-dien-1-one (**1a**): Yield 58%, yellow solid. ^{1}H NMR (500 MHz, $CDCl_3$), δ 7.53 (d, *J* = 2.0 Hz, 1H), 7.46-7.45 (m, 4H), 7.41-7.39 (m, 1H), 7.19 (s, 1H), 7.02 (d, *J* = 2.0 Hz, 1H), 1.34 (s, 9H), 1.30 (s, 9H); ^{13}C NMR (125 MHz, $CDCl_3$), δ 186.6, 149.4, 147.8, 142.5, 136.0, 135.1, 132.0, 130.4, 129.1, 128.8, 127.8, 124.9, 119.6, 35.5, 35.0, 30.30, 29.6, 29.5.

2,6-di-*tert*-butyl-4-(2-methoxybenzylidene)cyclohexa-2,5-dien-1-one (**1b**): Yield 42%, yellow solid. ^{1}H NMR (500 MHz, $CDCl_3$), δ 7.46 (d, *J* = 2.0 Hz, 1H), 7.40-7.37 (m, 3H), 7.07 (d, *J* = 2.5 Hz, 1H), 7.02 (t, *J* = 7.5 Hz, 1H), 6.95 (d, *J* = 8.0 Hz, 1H), 3.90 (s, 3H), 1.33 (s, 9H), 1.28 (s, 9H); ^{13}C NMR (125 MHz, $CDCl_3$), δ 186.7, 158.3, 149.0, 147.4, 138.7, 135.3, 131.8, 131.6, 130.8, 128.3, 124.9, 120.6, 110.8, 55.6, 35.4, 35.0, 29.6, 29.5.

2,6-di-*tert*-butyl-4-(3-methoxybenzylidene)cyclohexa-2,5-dien-1-one (**1c**): Yield 53%, yellow solid. ^{1}H NMR (500 MHz, CDCl3), δ 7.55 (d, *J* = 2.0 Hz, 1H), 7.35 (t, *J* = 8.0 Hz, 1H), 7.16 (s, 1H), 7.04 (d, *J* = 7.5 Hz, 1H), 7.01 (d, *J* = 2.5 Hz, 1H), 6.99 (s, 1H), 6.94 (d, *J* = 8.5 Hz, 1H), 3.85 (s, 3H), 1.33 (s, 9H), 1.30 (s, 9H); ^{13}C NMR (125 MHz, $CDCl_3$), δ 186.6, 159.8, 149.4, 147.9, 142.3, 137.2, 135.1, 132.1, 129.8, 127.8, 123.0, 115.2, 115.2, 55.3, 35.5, 35.0, 29.6, 29.5.

2,6-di-*tert*-butyl-4-(4-methoxybenzylidene)cyclohexa-2,5-dien-1-one (**1d**): Yield 65%, yellow solid. ^{1}H NMR (500 MHz, $CDCl_3$), δ 7.55 (d, *J* = 2.0 Hz, 1H), 7.44 (d, *J* = 9.0 Hz, 2H), 7.13 (s, 1H), 7.00 (d, *J* = 2.5 Hz, 1H), 6.99 (s, 1H), 6.98 (s, 1H), 3.88 (s, 3H), 1.33 (s, 9H), 1.32 (s, 9H); ^{13}C NMR (125 MHz, $CDCl_3$), δ 186.5, 160.6, 149.0, 147.2, 142.7, 135.4, 132.2, 130.5, 128.7, 127.8, 114.4, 55.4, 35.4, 35.0, 29.6, 29.5.

2,6-di-*tert*-butyl-4-(2-fluorobenzylidene)cyclohexa-2,5-dien-1-one (**1e**): Yield 50%, yellow solid. ^{1}H NMR (500 MHz, $CDCl_3$), δ 7.42 (t, *J* = 7.5 Hz, 1H), 7.40-7.39 (m, 2H), 7.24-7.22 (m, 2H), 7.18-7.14 (m, 1H), 7.04 (d, *J* = 2.5 Hz, 1H), 1.33 (s, 9H), 1.29 (s, 9H); ^{13}C NMR (125 MHz, $CDCl_3$), δ 186.6, 162.0 (d, *J* = 250 Hz), 149.7, 148.2, 134.8, 134.3 (d, *J* = 3.75 Hz), 133.1, 131.7, 131.0 (d, *J* = 7.5 Hz), 127.6, 124.2 (d, *J* = 3.75 Hz), 124.0 (d, *J* = 12.5 Hz), 116.1 (d, *J* = 22.5 Hz), 35.5, 35.1, 29.5, 29.5.

2,6-di-*tert*-butyl-4-(3-fluorobenzylidene)cyclohexa-2,5-dien-1-one (**1f**): Yield 50%,

yellow oil. ^{1}H NMR (500 MHz, $CDCl_3$), δ 7.47 (d, *J* = 2.5 Hz, 1H), 7.46-7.40 (m, 1H), 7.23 (d, *J* = 7.0 Hz, 1H), 7.16 (dt, *J* = 10 and 2.0 Hz, 1H), 7.11-7.07 (m, 3H), 7.00 (d, *J* = 2.5 Hz, 1H), 1.33 (s, 9H), 1.30 (s, 9H); ^{13}C NMR (125 MHz, $CDCl_3$), δ 186.6, 163.8 (d, *J* = 246.25 Hz), 149.9, 148.3, 140.4, 138.0 (d, *J* = 8.75 Hz), 134.8, 132.8, 130.4 (d, *J* = 8.75 Hz), 127.3, 126.1, 116.9 (d, *J* = 22.5 Hz), 116.0 (d, *J* = 21.25 Hz), 35.5, 35.1, 29.5, 29.5.

2,6-di-*tert*-butyl-4-(4-fluorobenzylidene)cyclohexa-2,5-dien-1-one (**1g**): Yield 46%, yellow solid. ^{1}H NMR (500 MHz, $CDCl_3$), δ 7.45-7.43 (m, 3H), 7.13 (m, 3H), 7.00 (d, *J* = 2.0 Hz, 1H), 1.33 (s, 9H), 1.30 (s, 9H); ^{13}C NMR (125 MHz, $CDCl_3$), δ 186.54, 164.1 (d, *J* = 250 Hz), 149.6, 147.9, 141.0, 135.0, 132.2 (d, *J* = 8.75 Hz), 132.1 (d, *J* = 6.0 Hz), 127.4, 116.1 (d, *J* = 22.5 Hz), 35.5, 35.0, 29.5, 29.5.

2,6-di-*tert*-butyl-4-(2-(trifluoromethyl)benzylidene)cyclohexa-2,5-dien-1-one (**1h**): Yield 52%, yellow solid. ^{1}H NMR (500 MHz, $CDCl_3$), δ 7.77 (d, *J* = 8.0 Hz, 1H), 7.60 (t, *J* = 7.5 Hz, 1H), 7.49 (t, *J* = 7.5 Hz, 1H), 7.42 (d, *J* = 8.0 Hz, 1H), 7.37 (d, *J* = 2.0 Hz, 1H), 7.17 (d, *J* = 2.0 Hz, 1H), 7.04 (d, *J* = 2.0 Hz, 1H), 1.34 (s, 9H), 1.25 (s, 9H); ^{13}C NMR (125 MHz, $CDCl_3$), δ 186.7, 149.9, 148.5 137.5, 134.5, 134.1, 133.6, 132.5, 131.5, 129.6 (q, *J* = 30 Hz), 128.7, 127.4, 126.5 (q, *J* = 5.0 Hz), 125.1 (q, *J* = 272.5 Hz), 35.43, 35.10, 29.49, 29.47.

2,6-di-*tert*-butyl-4-(3-(trifluoromethyl)benzylidene)cyclohexa-2,5-dien-1-one (**1i**): Yield 48%, yellow solid. ^{1}H NMR (500 MHz, ($CDCl_3$), δ 7.71 (s, 1H), 7.64 (d, *J* = 7.0, 1H), 7.62-7.58 (m, 2H), 7.41 (d, *J* = 2.5 Hz, 1H), 7.17 (s, 1H), 7.01 (d, *J* = 2.0 Hz, 1H), 1.33 (s, 9H), 1.29 (s, 9H); ^{13}C NMR (125 MHz, $CDCl_3$), δ 186.5, 150.2, 148.6, 139.6, 136.6, 134.6, 133.2, 133.2, 131.7 (q, *J* = 32.5 Hz), 129.3, 127.1, 127.0 (q, *J* = 3.75 Hz), 125.4 (q, *J* = 3.75 Hz), 124.9 (q, *J* = 271.25 Hz), 35.51, 35.10, 29.50, 29.46.

2,6-di-*tert*-butyl-4-(4-(trifluoromethyl)benzylidene)cyclohexa-2,5-dien-1-one (**1j**): Yield 60%, yellow solid. ^{1}H NMR (500 MHz, $CDCl_3$), δ 7.70 (d, *J* = 8.0 Hz, 2H), 7.54 (d, *J* = 8.5 Hz, 2H), 7.41 (d, *J* = 2.0 Hz, 1H), 7.16 (s, 1H), 7.01 (d, J = 2.0 Hz, 1H), 1.33 (s, 9H), 1.29 (s, 9H); ^{13}C NMR (125 MHz, $CDCl_3$), δ 186.5, 150.2, 148.6, 139.7, 139.4, 134.7, 133.5, 130.7 (q, *J* = 32.5 Hz), 130.4, 127.2, 125.7 (q, *J* = 3.75 Hz), 125.0 (q, *J* = 270 Hz), 35.53, 35.10, 29.53, 29.50.

2,6-di-*tert*-butyl-4-(2-(trifluoromethoxy)benzylidene)cyclohexa-2,5-dien-1-one (**1k**): Yield 54%, yellow solid. ^{1}H NMR (500 MHz, $CDCl_3$), δ 7.47-7.43 (m, 2H), 7.39-7.35 (m, 2H), 7.33 (d, *J* = 2.5 Hz, 1H), 7.23 (s, 1H), 7.05 (d, *J* = 2.5 Hz, 1H), 1.34 (s, 9H), 1.28 (s, 9H); ^{13}C NMR (125 MHz, $CDCl_3$), δ 186.7, 149.9, 148.3, 147.8, 135.1, 134.6, 133.5, 132.3, 130.4, 129.1, 127.4, 126.7, 121.6 (q, *J* = 256.25 Hz), 121.1, 35.5 35.1, 29.5.

2,6-di-*tert*-butyl-4-(3-(trifluoromethoxy)benzylidene)cyclohexa-2,5-dien-1-one (**1l**): Yield 56%, yellow solid. ^{1}H NMR (500 MHz, $CDCl_3$), δ 7.50 (t, *J* = 7.5 Hz, 1H), 7.44 (d, *J* = 2.0 Hz, 1H), 7.37 (d, *J* = 8.0 Hz, 1H), 7.32 (s, 1H), 7.25 (d, *J* = 8.0 Hz, 1H), 7.13 (s, 1H), 7.01 (d, *J* = 2.0 Hz, 1H), 1.33 (s, 8H), 1.29 (s, 9H); ^{13}C NMR (125 MHz, $CDCl_3$), δ 186.5, 150.2, 149.5, 148.5, 139.8, 137.8, 134.7, 133.1, 130.2, 128.5, 127.1, 122.3, 121.5 (q, *J* = 256.25 Hz), 121.3, 35.5, 35.1, 29.5, 29.5.

2,6-di-*tert*-butyl-4-(4-(trifluoromethoxy)benzylidene)cyclohexa-2,5-dien-1-one (**1m**): Yield 58%, yellow solid. ^{1}H NMR (500 MHz, $CDCl_3$), δ 7.49 (d, *J* = 8.5 Hz, 2H), 7.44 (d, *J* = 2.0 Hz, 1H), 7.31 (d, *J* = 8.0 Hz, 2H), 7.13 (s, 1H), 7.01 (d, *J* = 2.5 Hz, 1H), 1.33 (s, 9H), 1.30 (s, 9H); ^{13}C NMR (125 MHz, $CDCl_3$), δ 186.5, 149.9, 149.5, 148.2, 140.2, 134.8, 134.5, 132.6, 131.7, 127.1, 121.5 (q, *J* = 256.25 Hz), 121.0, 35.50, 35.06, 29.54, 29.50.

2,6-di-*tert*-butylcyclohexa-4-(2-bromobenzylidene)-2,5-dien-1-one (**1n**): Yield 65%, yellow solid. ^{1}H NMR (500 MHz, $CDCl_3$), δ 7.68 (d, *J* = 8.0 Hz, 1H), 7.39 (m, 2H), 7.28-7.27 (m, 1H), 7.26 (s, 1H), 7.23 (s, 1H), 7.07 (d, *J* = 2.5 Hz, 1H), 1.34 (s, 9H), 1.27 (s, 9H); ^{13}C NMR (125 MHz, $CDCl_3$), δ 186.6, 149.7, 148.3, 140.8, 135.9, 134.6, 133.2, 132.6, 132.3, 130.3, 127.7, 127.2, 125.1, 35.5 35.1, 29.5.

2,6-di-*tert*-butylcyclohexa-4-(3-bromobenzylidene)-2,5-dien-1-one (**1o**): Yield 64%, yellow oil. ^{1}H NMR (500 MHz, $CDCl_3$), δ 7.60 (s, 1H), 7.52 (d, *J* = 8.0 Hz, 1H), 7.44 (d, *J* = 2.5 Hz, 1H), 7.38 (d, *J* = 7.5 Hz, 1H), 7.34 (t, *J* = 7.5 Hz, 1H), 7.08 (s, 1H), 6.99 (d, *J* = 2.3 Hz, 1H), 1.33 (s, 9H), 1.30 (s, 9H); ^{13}C NMR (125 MHz, $CDCl_3$), δ 186.5, 150.0, 148.4, 139.9, 137.9, 134.7, 133.0, 133.0, 131.8, 130.2, 128.7, 127.2, 122.8, 35.5, 35.1, 29.5, 29.5.

2,6-di-*tert*-butylcyclohexa-4-(4-bromobenzylidene)-2,5-dien-1-one (**1p**): Yield 54%,

yellow solid. ^{1}H NMR (500 MHz, $CDCl_3$), δ 7.58 (d, *J* = 8.5 Hz, 2H), 7.43 (d, *J* = 2.5 Hz, 1H), 7.31 (d, *J* = 8.5 Hz, 2H), 7.08 (s, 1H), 6.99 (d, *J* = 2.5 Hz, 1H), 1.33 (s, 9H), 1.30 (s, 9H); ^{13}C NMR (125 MHz, $CDCl_3$), δ 186.5, 149.8, 148.1, 140.6, 134.8, 134.8, 132.4, 132.0, 131.7, 127.2, 123.5, 35.5, 35.1, 29.5, 29.5.

2,6-di-*tert*-butyl-4-(2-chlorobenzylidene)cyclohexa-2,5-dien-1-one (**1q**): Yield 63%, yellow solid. ^{1}H NMR (500 MHz, $CDCl_3$), δ 7.49-7.48 (m, 1H), 7.42-7.40 (m, 1H), 7.35-7.33 (m, 2H), 7.31 (d, *J* = 2.5 Hz, 1H), 7.30 (s, 1H), 7.08 (d, *J* = 2.5 Hz, 1H), 1.34 (s, 9H), 1.27 (s, 9H); ^{13}C NMR (125 MHz, $CDCl_3$), δ 186.7, 149.8, 148.3, 138.6, 134.9, 134.7, 134.1, 132.9, 132.2, 130.2, 130.1, 127.7, 126.7, 35.5, 35.1, 29.5.

2,6-di-*tert*-butyl-4-(3-chlorobenzylidene)cyclohexa-2,5-dien-1-one (**1r**): Yield 57%, yellow oil. ^{1}H NMR (500 MHz, $CDCl_3$), δ 7.44-7.43 (m, 2H), 7.40-7.35 (m, 2H), 7.33-7.31 (m, 1H), 7.09 (s, 1H), 6.99 (d, *J* = 2.0 Hz, 1H), 1.33 (s, 9H), 1.30 (s, 9H); ^{13}C NMR (125 MHz, $CDCl_3$), δ 186.5, 149.9, 148.4, 140.0, 137.7, 134.8, 134.7, 132.9, 130.1, 130.0, 128.9, 128.3, 127.2, 35.5, 35.1, 29.5, 29.5.

2,6-di-*tert*-butyl-4-(4-chlorobenzylidene)cyclohexa-2,5-dien-1-one (**1s**): Yield 52%, yellow solid. ^{1}H NMR (500 MHz, $CDCl_3$), δ 7.44-7.42 (m, 3H), 7.39 (d, *J* = 8.5 Hz, 2H), 7.11 (s, 1H), 7.00 (d, *J* = 2.5 Hz, 1H), 1.33 (s, 9H), 1.29 (s, 9H); ^{13}C NMR (125 MHz, $CDCl_3$), δ 186.5, 149.8, 148.1, 140.6, 135.2, 134.9, 134.4, 132.4, 131.5, 129.1, 127.2, 35.5, 35.1, 29.5, 29.5.

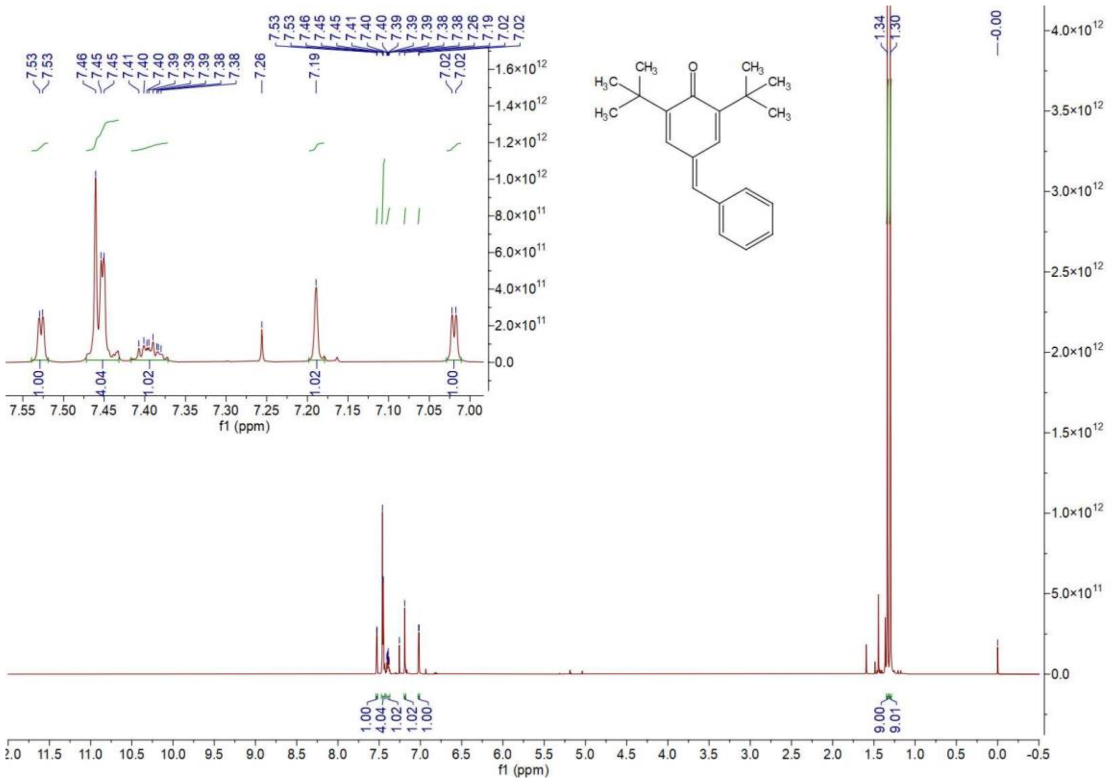
7.53
7.53
7.46
7.45
7.45
7.41
7.40
7.40
7.39
7.39
7.39
7.38
7.38
7.26
7.19
7.02
7.02
1.34
1.30
0.00
1.00
4.04
1.02
1.02
1.00
9.00
9.01
f1 (ppm)

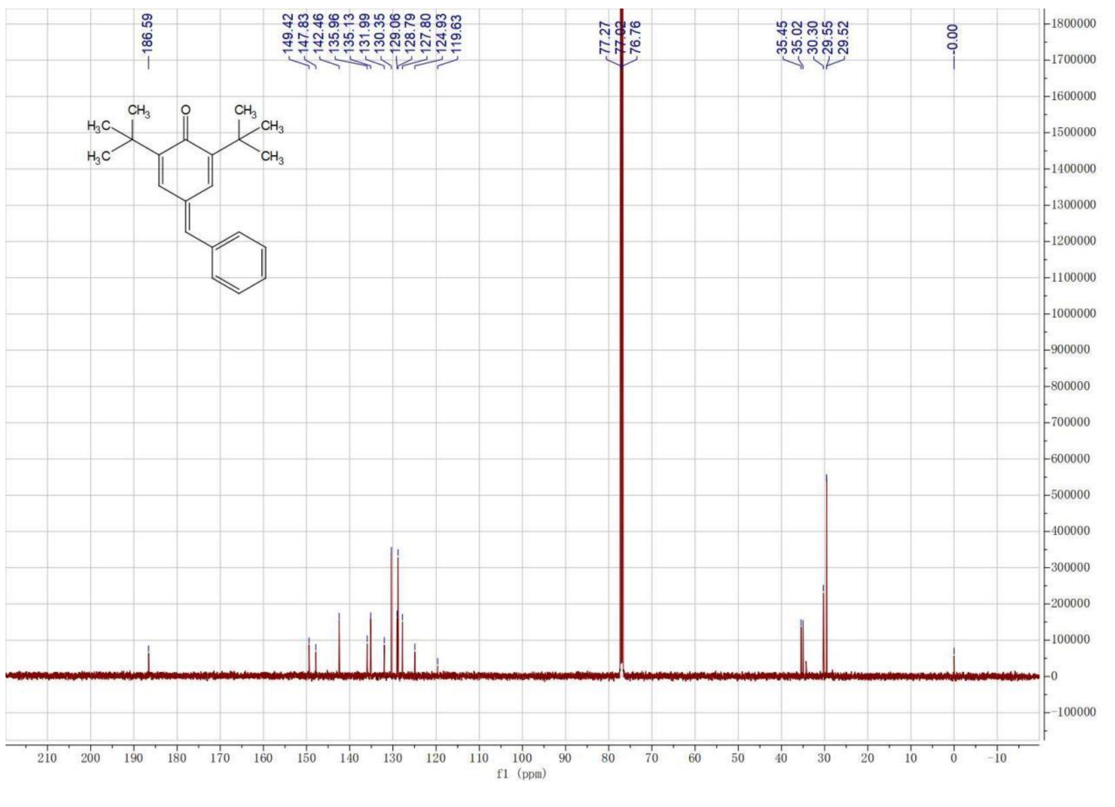
186.59
149.42
147.83
142.46
135.96
135.13
131.99
130.35
129.06
128.79
127.80
124.93
119.63
77.27
77.02
76.76
35.45
35.02
30.30
29.55
29.52
-0.00
f1 (ppm)

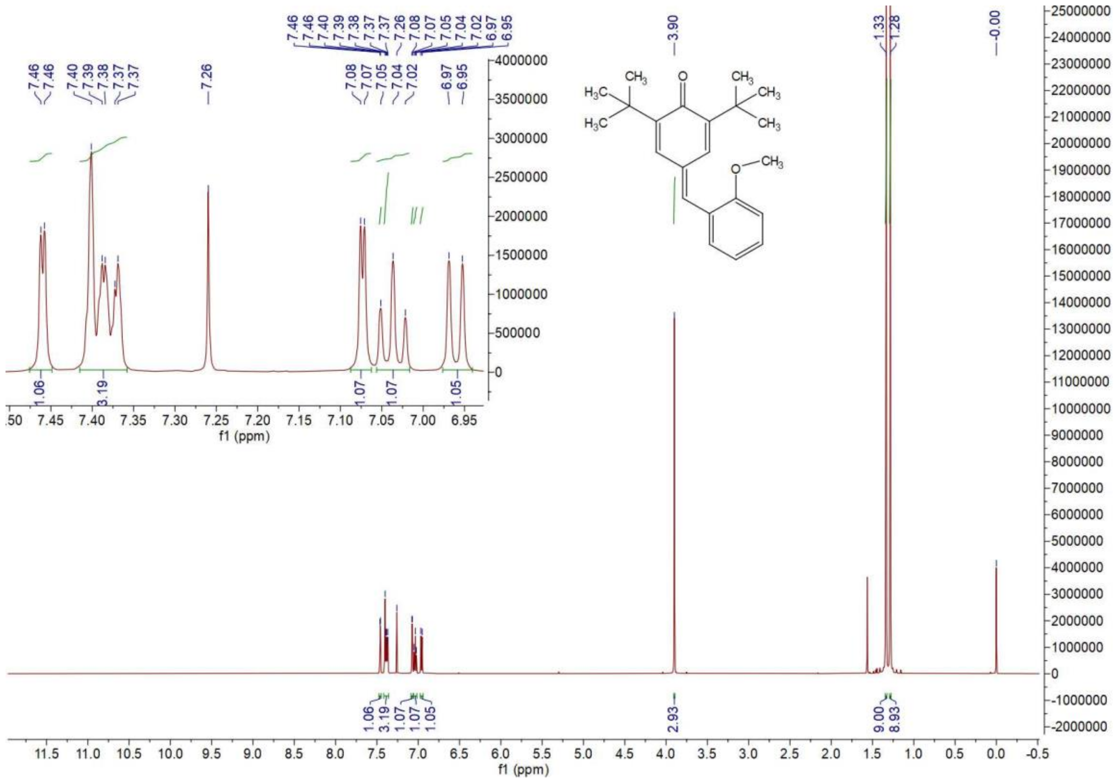
7.46
7.46
7.40
7.39
7.38
7.37
7.37
7.26
7.08
7.07
7.05
7.04
7.02
6.97
6.95
3.90
1.33
1.28
-0.00
1.06
3.19
1.07
1.07
1.05
2.93
9.00
8.93
f1 (ppm)

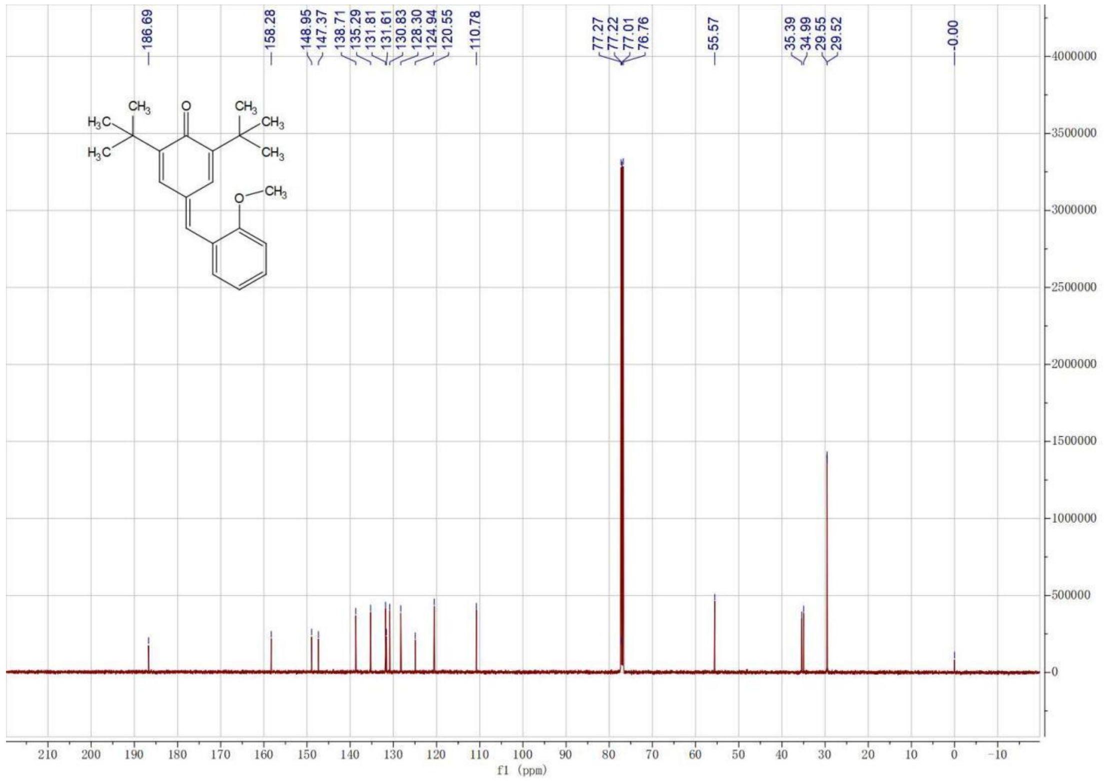
186.69
158.28
148.95
147.37
138.71
135.29
131.81
131.61
130.83
128.30
124.94
120.55
110.78
77.27
77.22
77.01
76.76
55.57
35.39
34.99
29.55
29.52
-0.00
f1 (ppm)

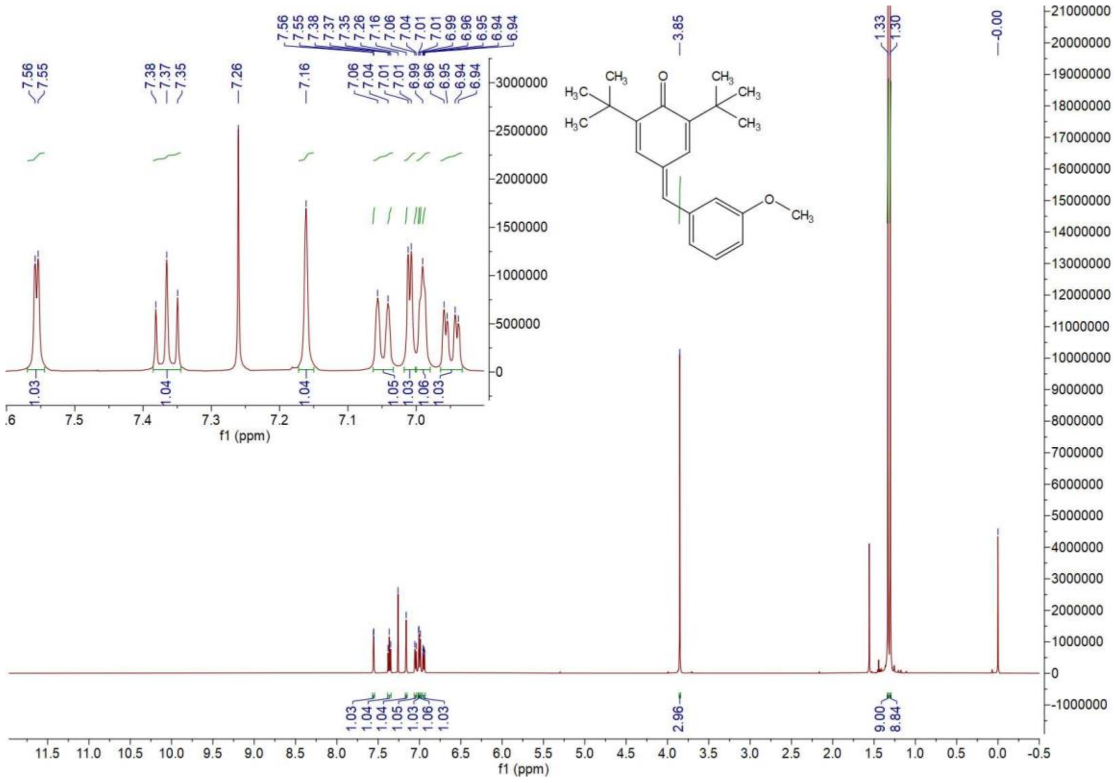
7.56
7.55
7.38
7.37
7.35
7.26
7.16
7.06
7.04
7.01
7.01
6.99
6.96
6.95
6.94
3.85
1.33
1.30
0.00
1.03
1.04
1.04
1.05
1.03
1.06
1.03
2.96
9.00
8.84
f1 (ppm)

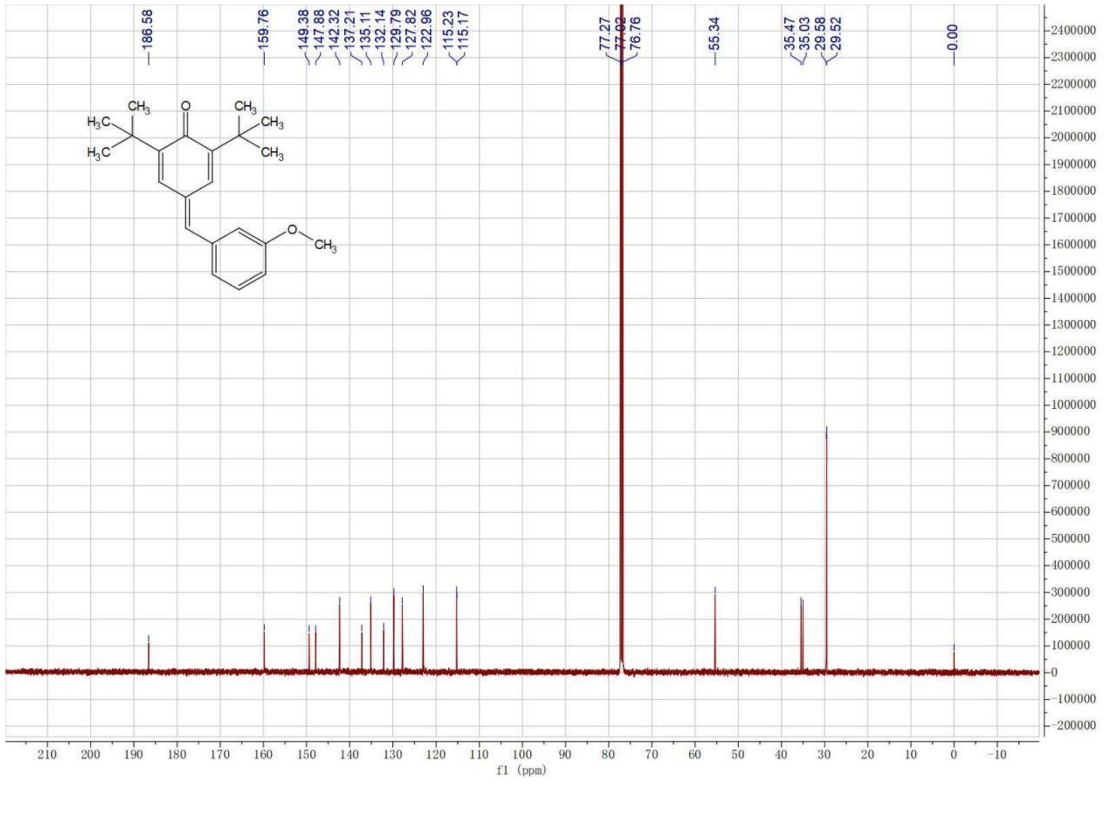
186.58
159.76
149.38
147.88
142.32
137.21
135.11
132.14
129.79
127.82
122.96
115.23
115.17
77.27
77.02
76.76
55.34
35.47
35.03
29.58
29.52
0.00
f1 (ppm)

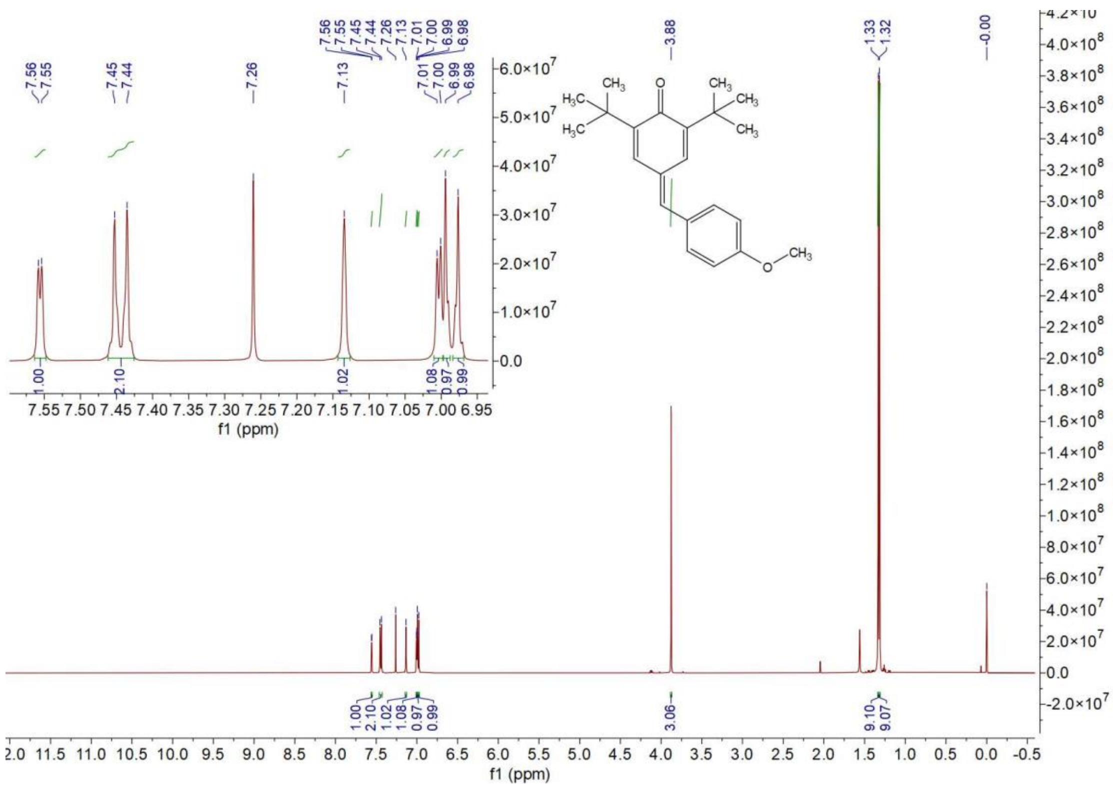
7.56
7.55
7.45
7.44
7.26
7.13
7.01
7.00
6.99
6.98
3.88
1.33
1.32
0.00
1.00
2.10
1.02
1.08
0.97
0.99
3.06
9.10
9.07
f1 (ppm)
H$_3$C
CH$_3$
O
CH$_3$
CH$_3$
H$_3$C
CH$_3$
O
CH$_3$

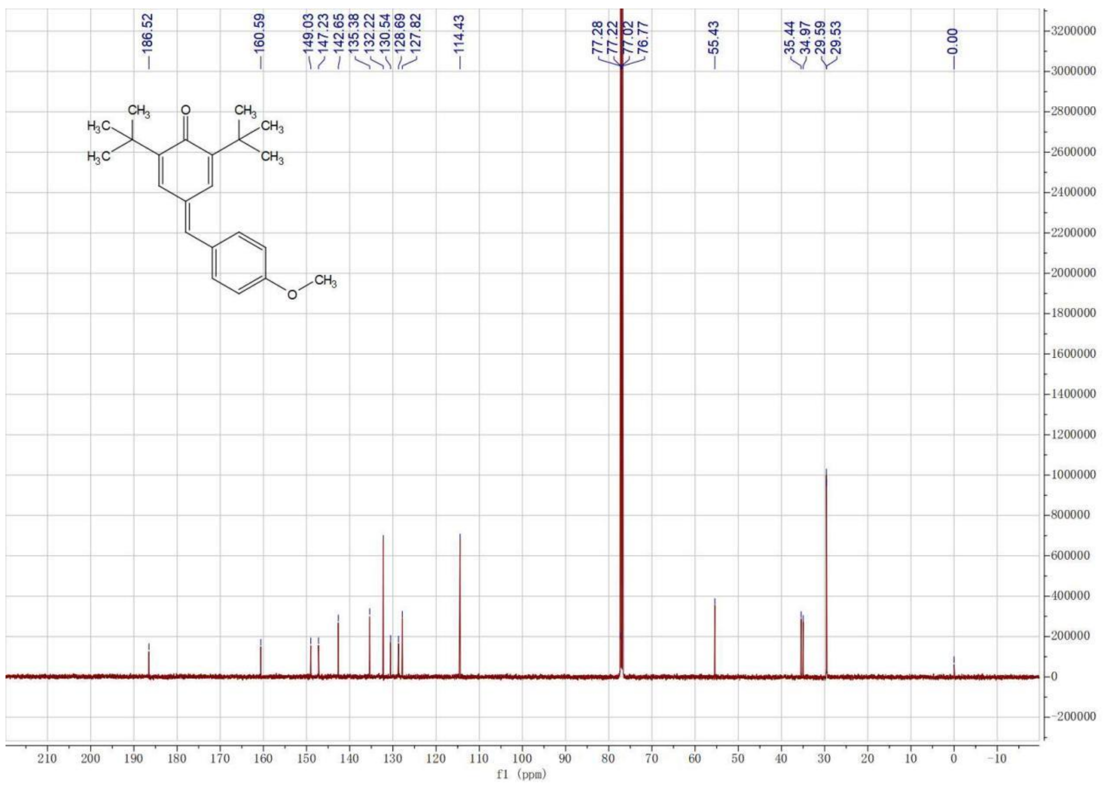
186.52
160.59
149.03
147.23
142.65
135.38
132.22
130.54
128.69
127.82
114.43
77.28
77.22
77.02
76.77
55.43
35.44
34.97
29.59
29.53
0.00
f1 (ppm)
H$_3$C
CH$_3$
O
CH$_3$
CH$_3$
H$_3$C
CH$_3$
O
CH$_3$

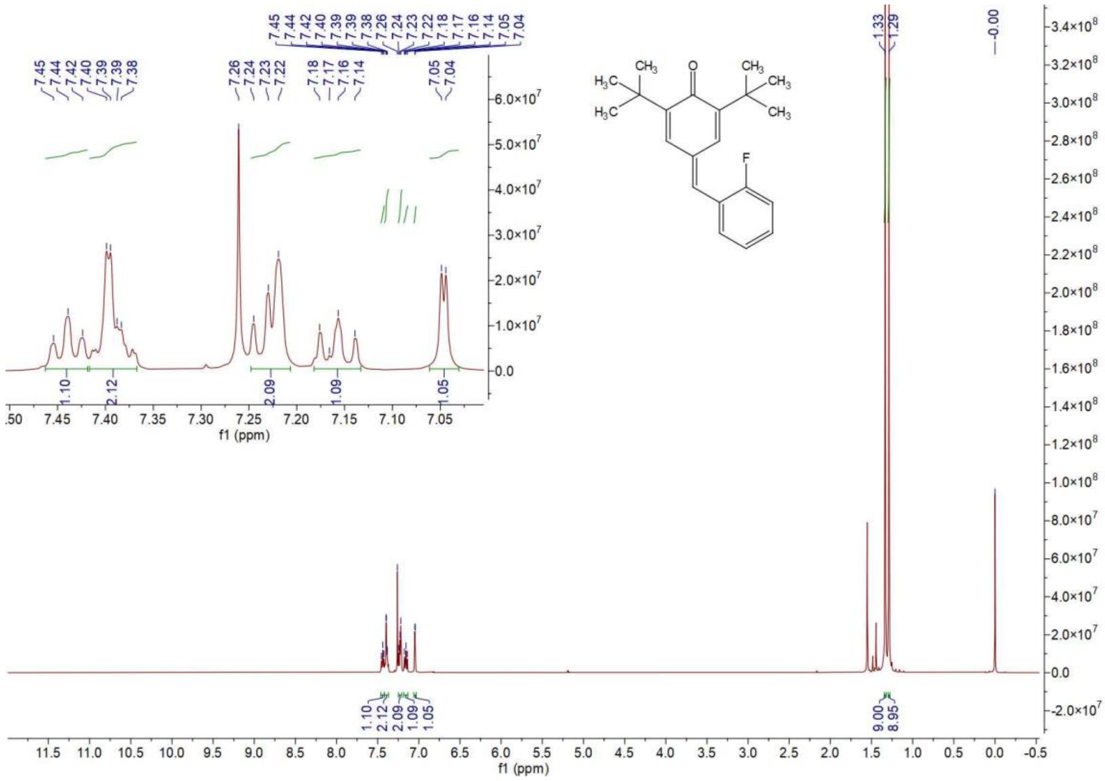
7.45 7.44 7.42 7.40 7.39 7.39 7.38 7.26 7.24 7.23 7.22 7.18 7.17 7.16 7.14 7.05 7.04
1.33 1.29
0.00
1.10 2.12 2.09 1.09 1.05
9.00 8.95
f1 (ppm)

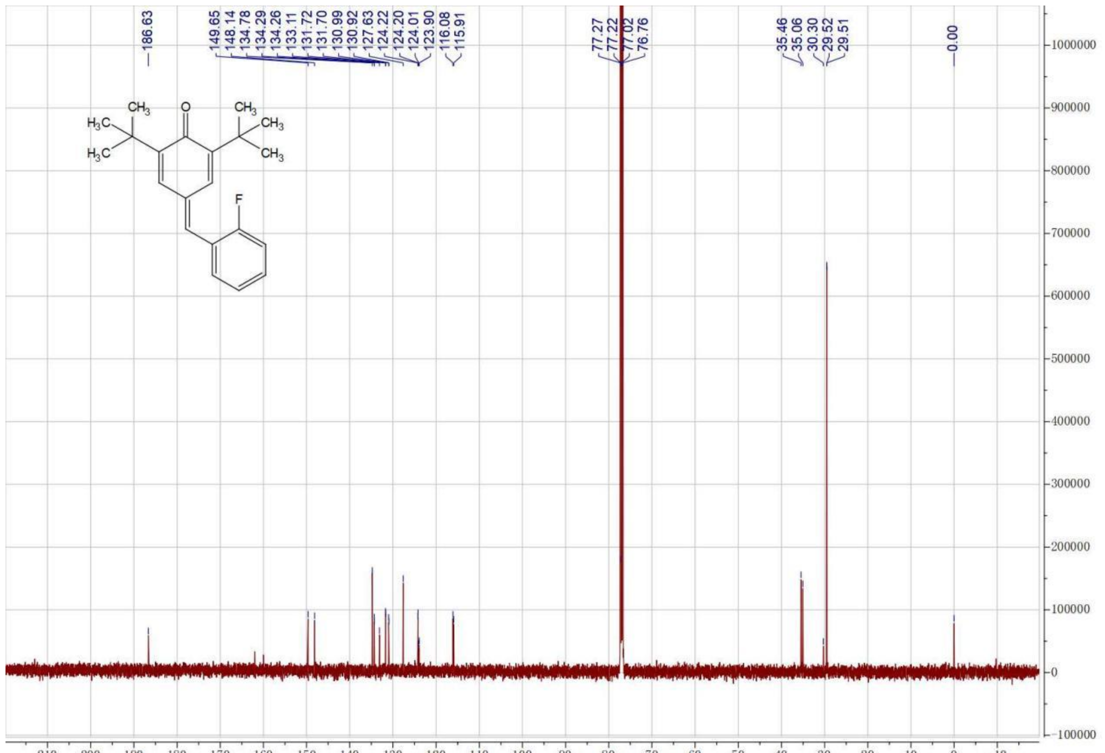
186.63
149.65 148.14 134.78 134.29 134.26 133.11 131.72 131.70 130.99 130.92 127.63 124.22 124.20 124.01 123.90 116.08 115.91
77.27 77.22 77.02 76.76
35.46 35.06 30.30 29.52 29.51
0.00
f1 (ppm)

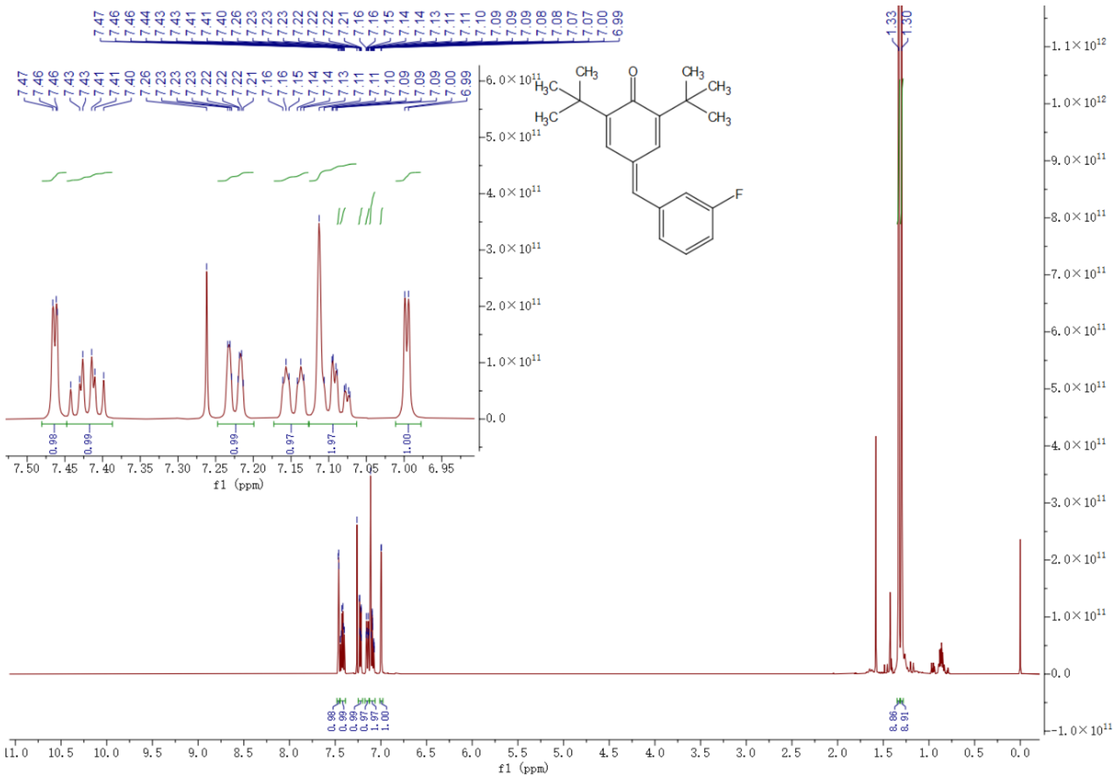
7.47 7.46 7.46 7.44 7.43 7.43 7.41 7.41 7.40 7.26 7.23 7.23 7.23 7.22 7.22 7.22 7.22 7.21 7.16 7.16 7.15 7.14 7.14 7.13 7.11 7.11 7.10 7.09 7.09 7.09 7.08 7.07 7.07 7.00 6.99
1.33 1.30
0.98 0.99 0.99 0.97 1.97 1.00
8.86 8.91
f1 (ppm)

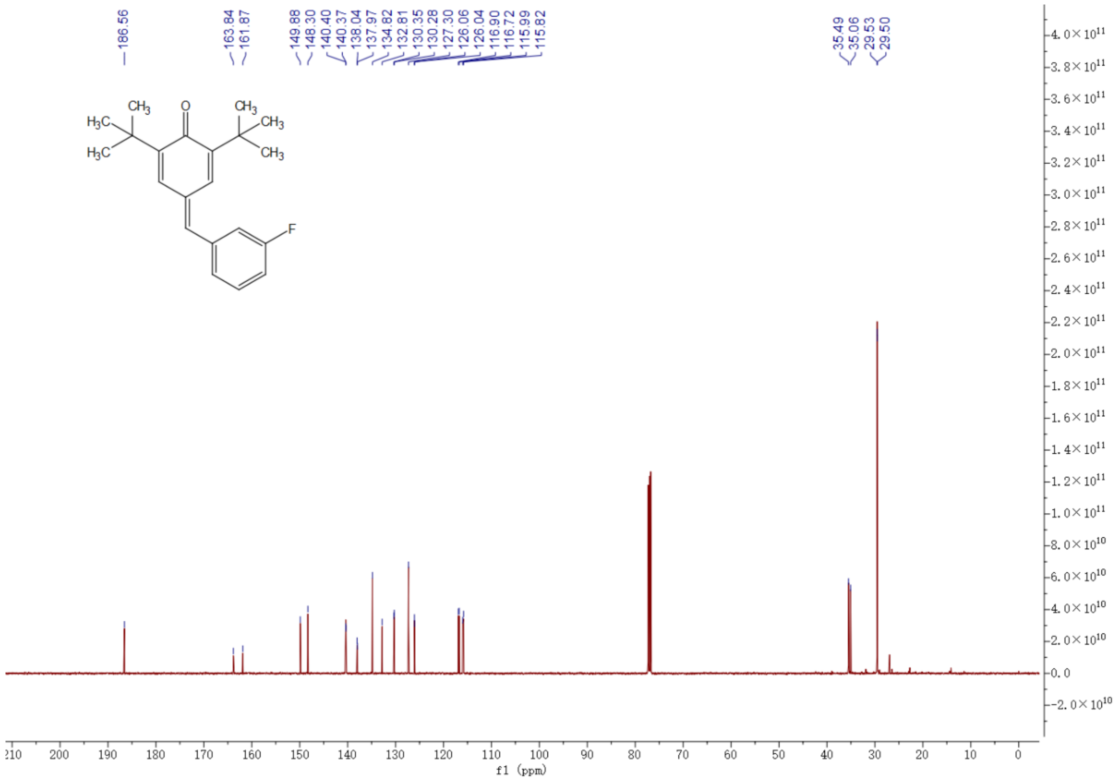
186.56
163.84 161.87
149.88 148.30 140.40 140.37 138.04 137.97 134.82 132.81 130.35 130.28 127.30 126.06 126.04 116.90 116.72 115.99 115.82
35.49 35.06 29.53 29.50
f1 (ppm)

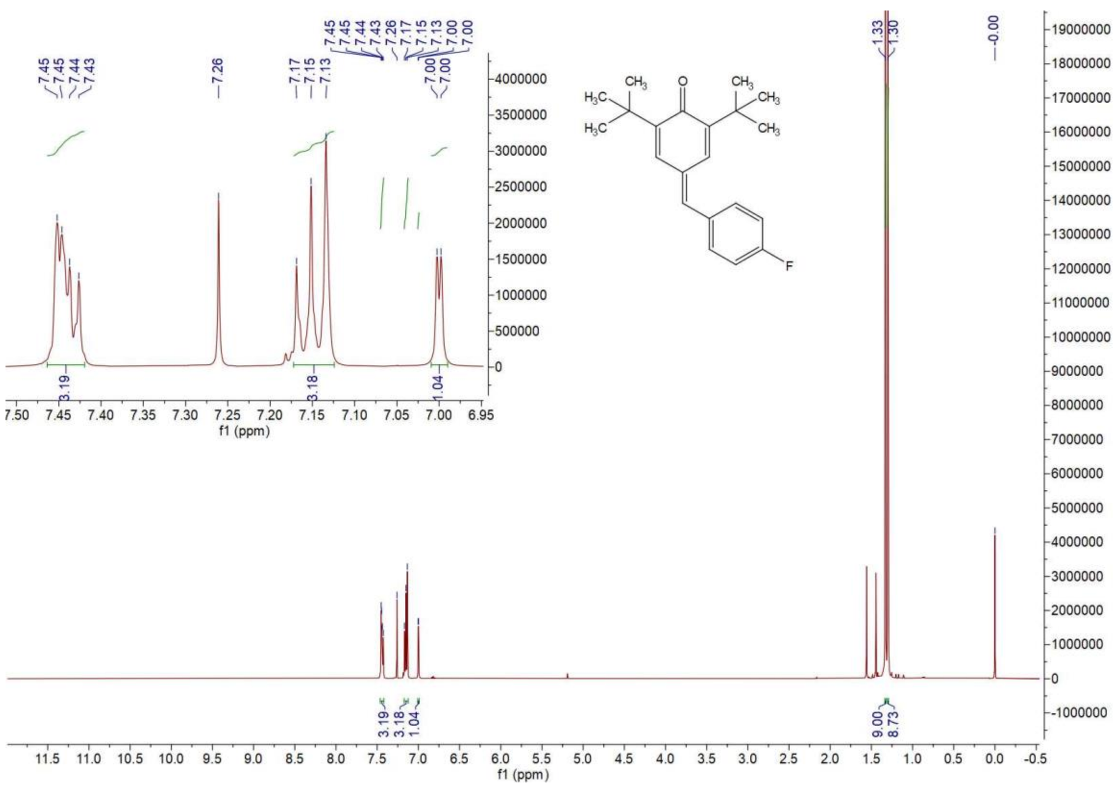
7.45
7.45
7.44
7.43
7.26
7.17
7.15
7.13
7.00
7.00
3.19
3.18
1.04
f1 (ppm)
1.33
1.30
0.00
9.00
8.73
F
O
f1 (ppm)

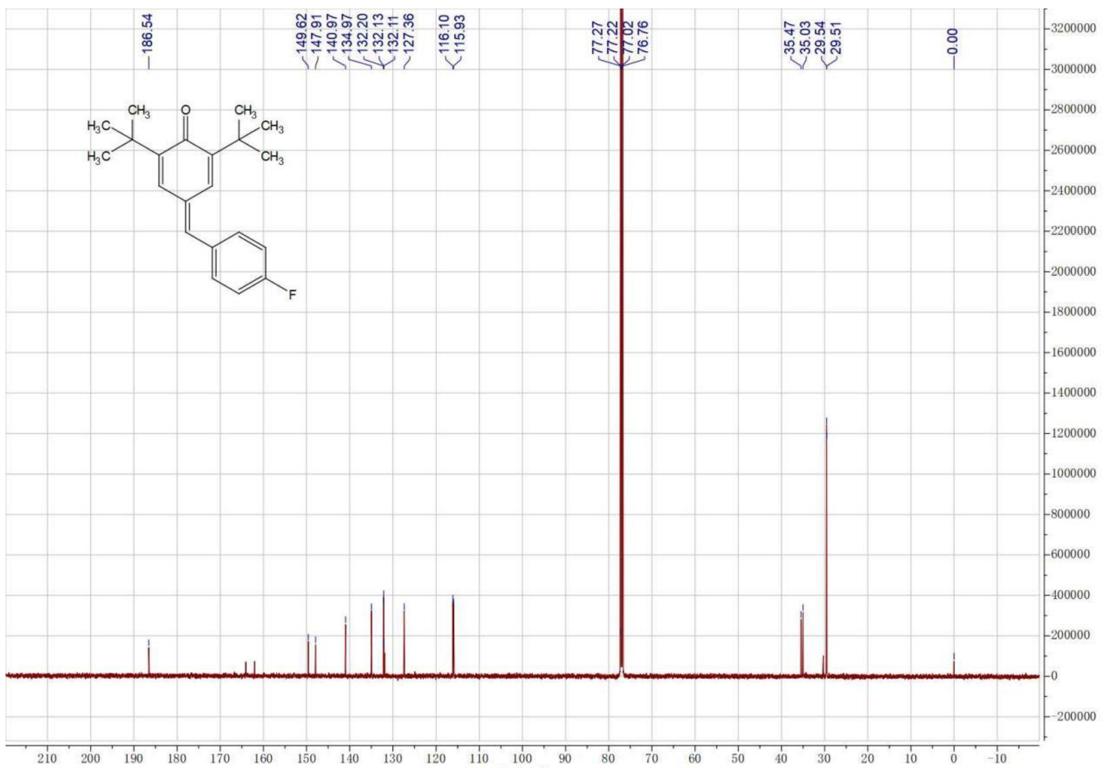
186.54
149.62
147.91
140.97
134.97
132.20
132.13
132.11
127.36
116.10
115.93
77.27
77.22
77.02
76.76
35.47
35.03
29.54
29.51
0.00
O
F
f1 (ppm)

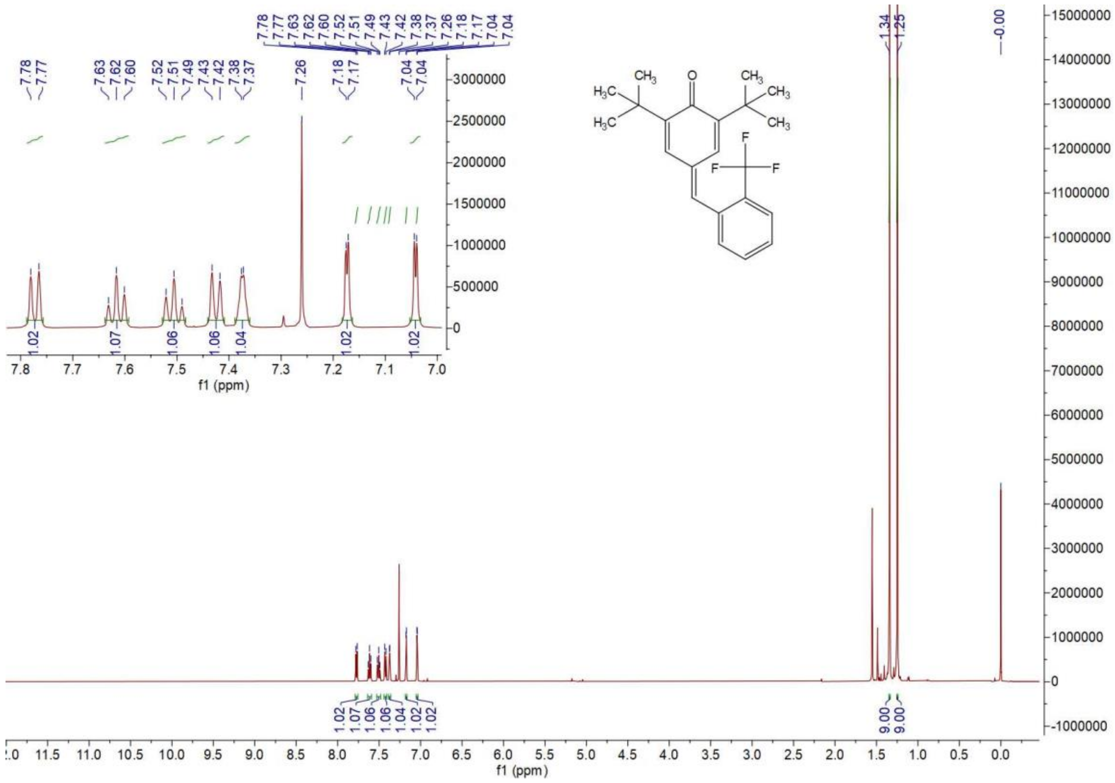
7.78
7.77
7.63
7.62
7.60
7.52
7.51
7.49
7.43
7.42
7.38
7.37
7.26
7.18
7.17
7.04
7.04
1.34
1.25
0.00
1.02
1.07
1.06
1.06
1.04
1.02
1.02
9.00
9.00
f1 (ppm)

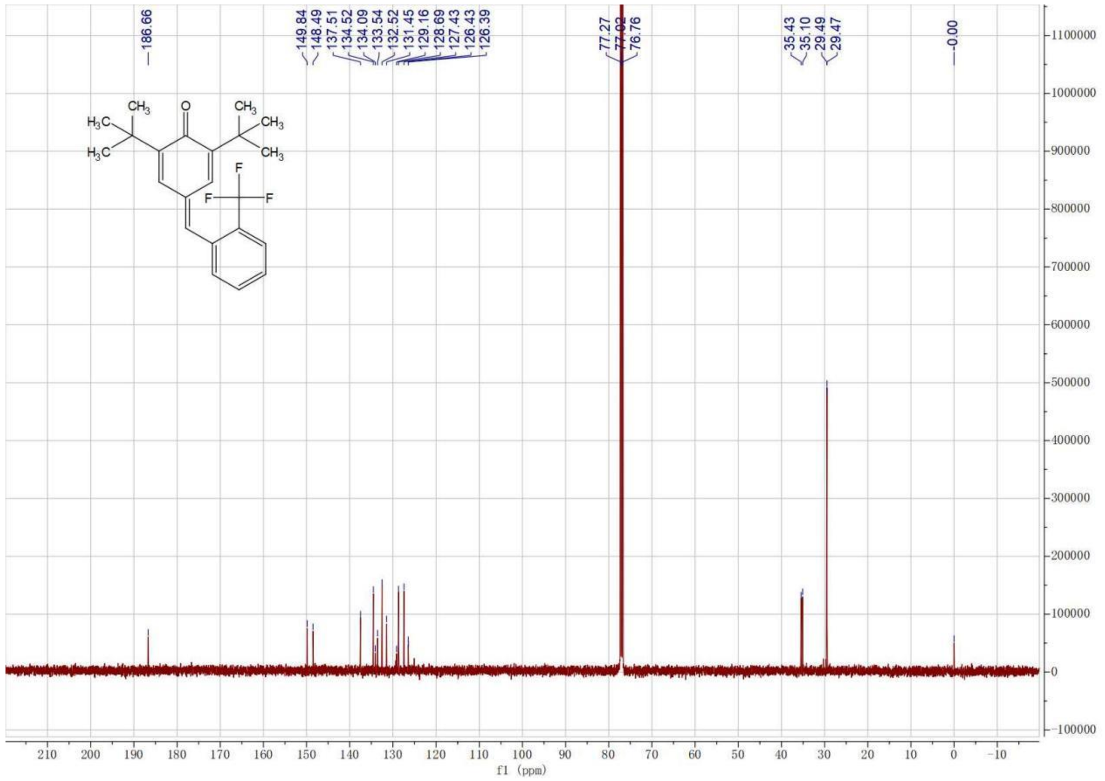
186.66
149.84
148.49
137.51
134.52
134.09
133.54
132.52
131.45
129.16
128.69
127.43
126.43
126.39
77.27
77.02
76.76
35.43
35.10
29.49
29.47
0.00
f1 (ppm)

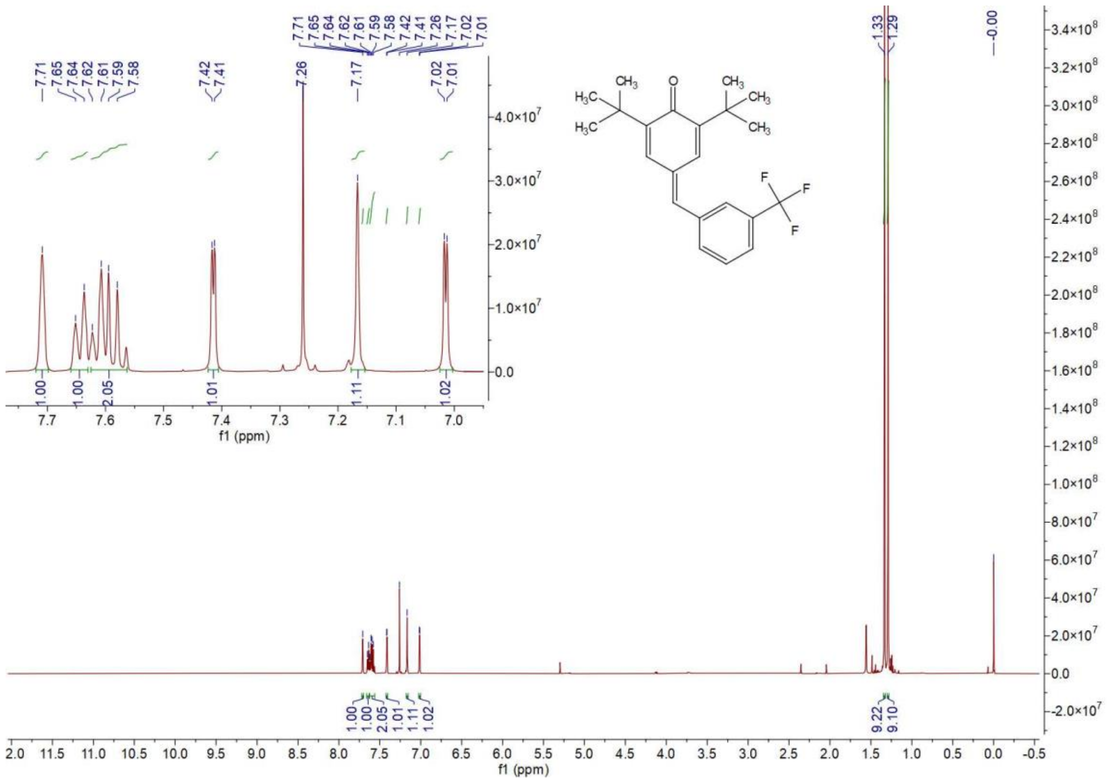
7.71 7.65 7.64 7.62 7.61 7.59 7.58 7.42 7.41 7.26 7.17 7.02 7.01
1.33 1.29
0.00
1.00 1.00 2.05 1.01 1.11 1.02
9.22 9.10
f1 (ppm)
F
F
F
O
H_3C
CH_3

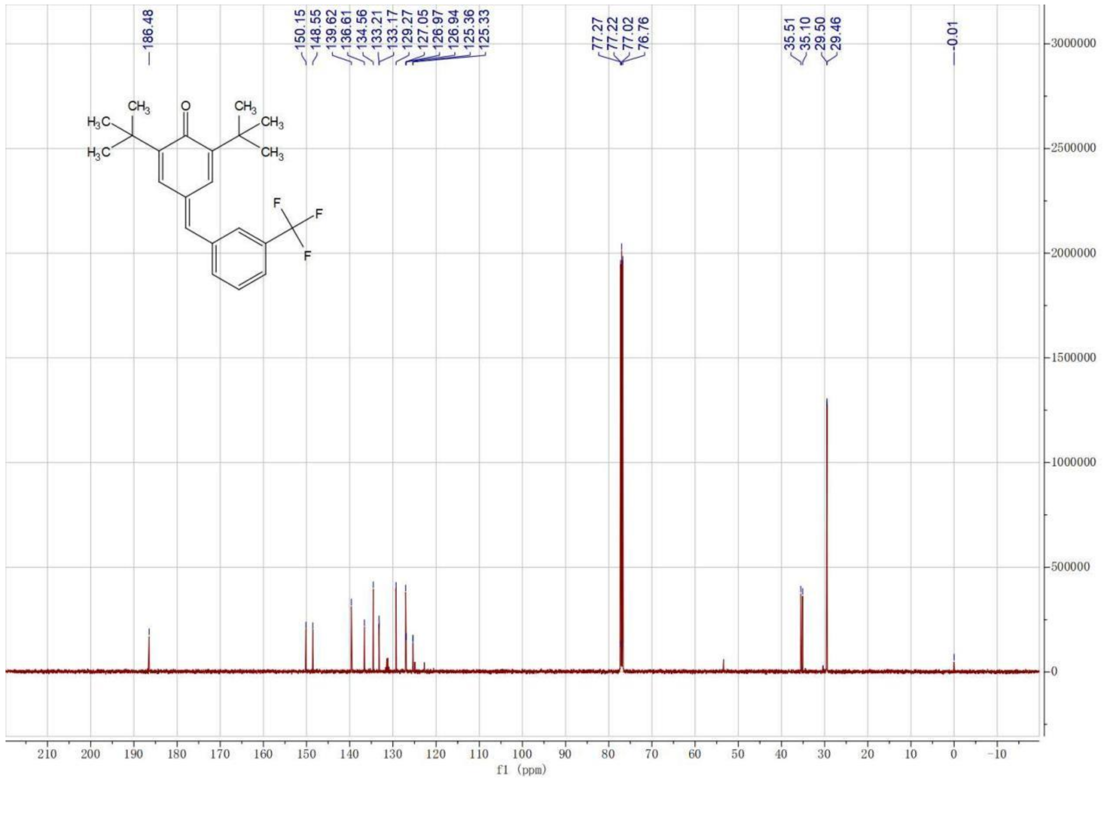
186.48
150.15 148.55 139.62 136.61 134.56 133.21 133.17 129.27 127.05 126.97 126.94 125.36 125.33
77.27 77.22 77.02 76.76
35.51 35.10 29.50 29.46
-0.01
f1 (ppm)
F
F
F
O
H_3C
CH_3

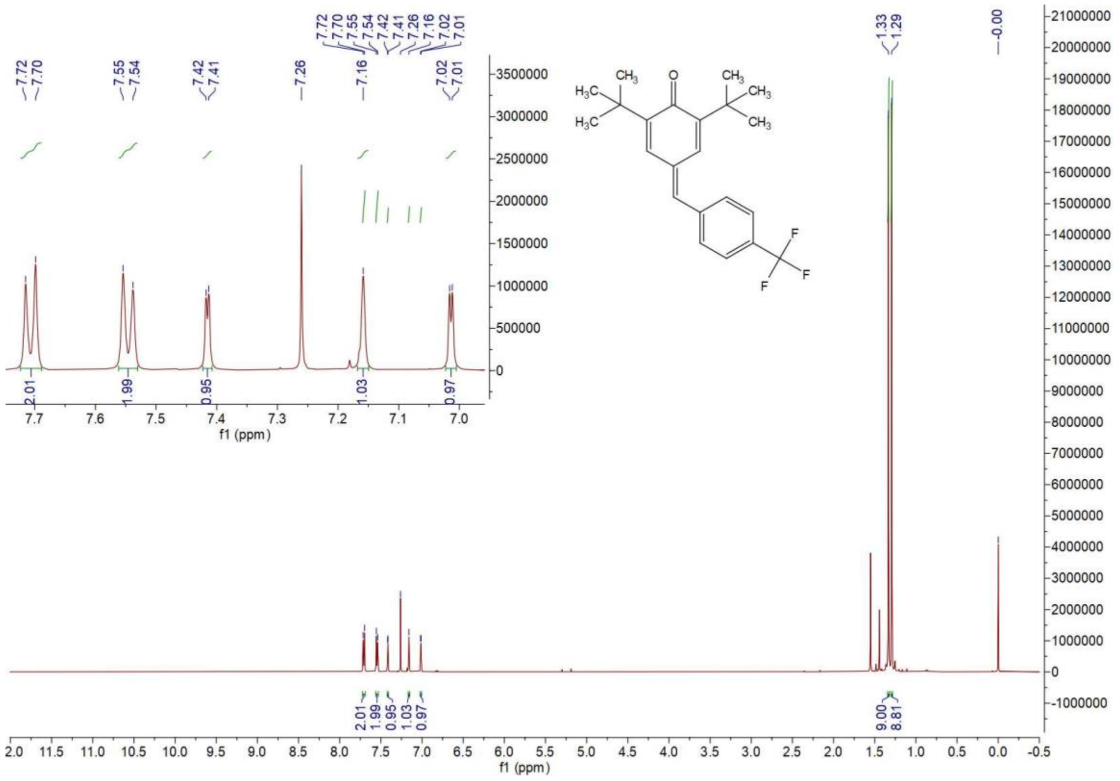
7.72 7.70 7.55 7.54 7.42 7.41 7.26 7.16 7.02 7.01
2.01 1.99 0.95 1.03 0.97
f1 (ppm)
1.33 1.29
0.00
9.00 8.81
F
F
F

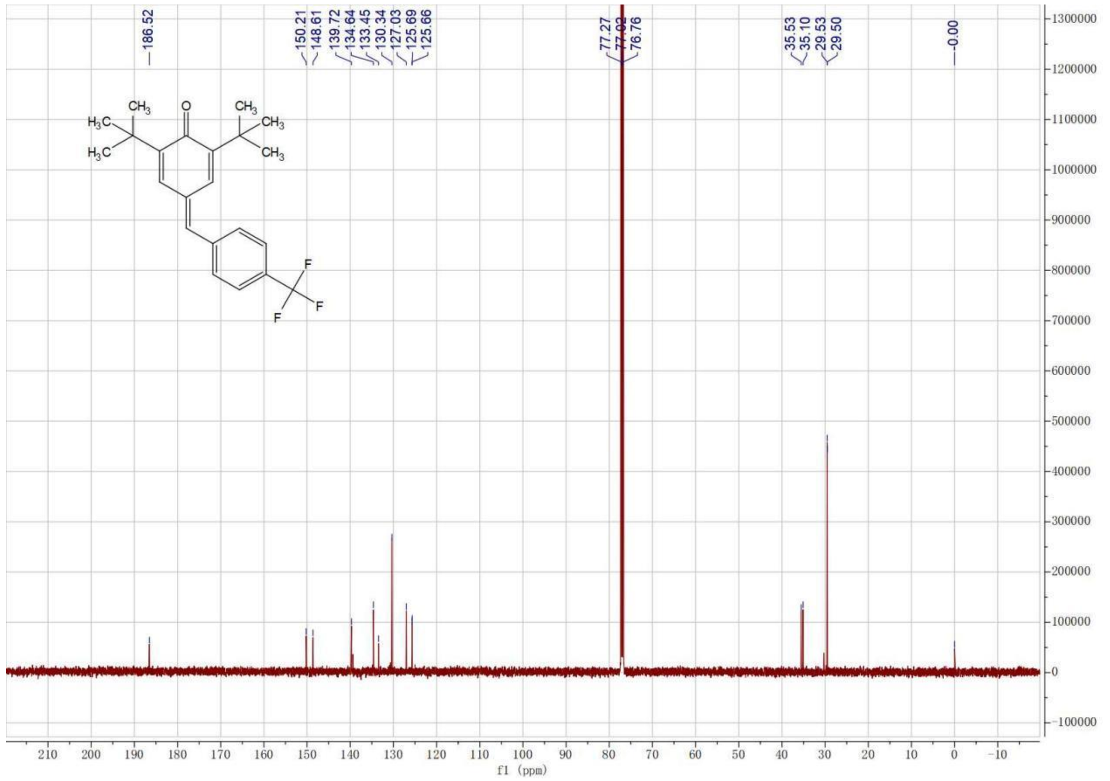
186.52
150.21 148.61 139.72 134.64 133.45 130.34 127.03 125.69 125.66
77.27 77.02 76.76
35.53 35.10 29.53 29.50
0.00
F
F
F
f1 (ppm)

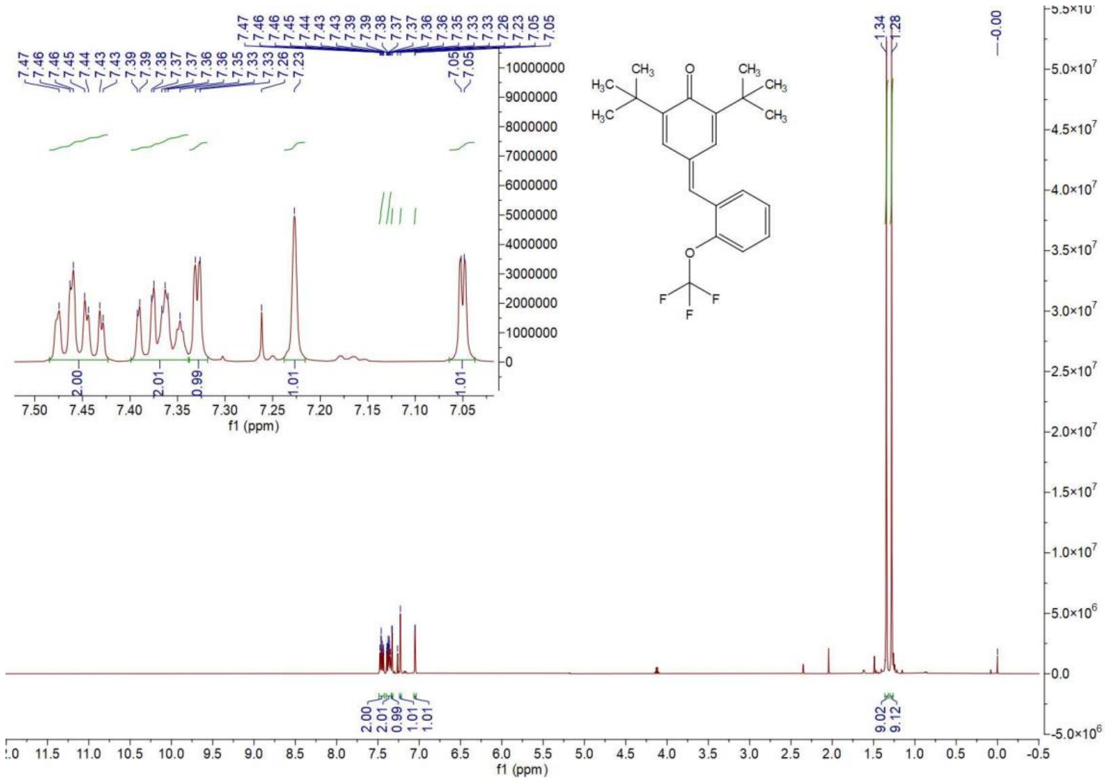

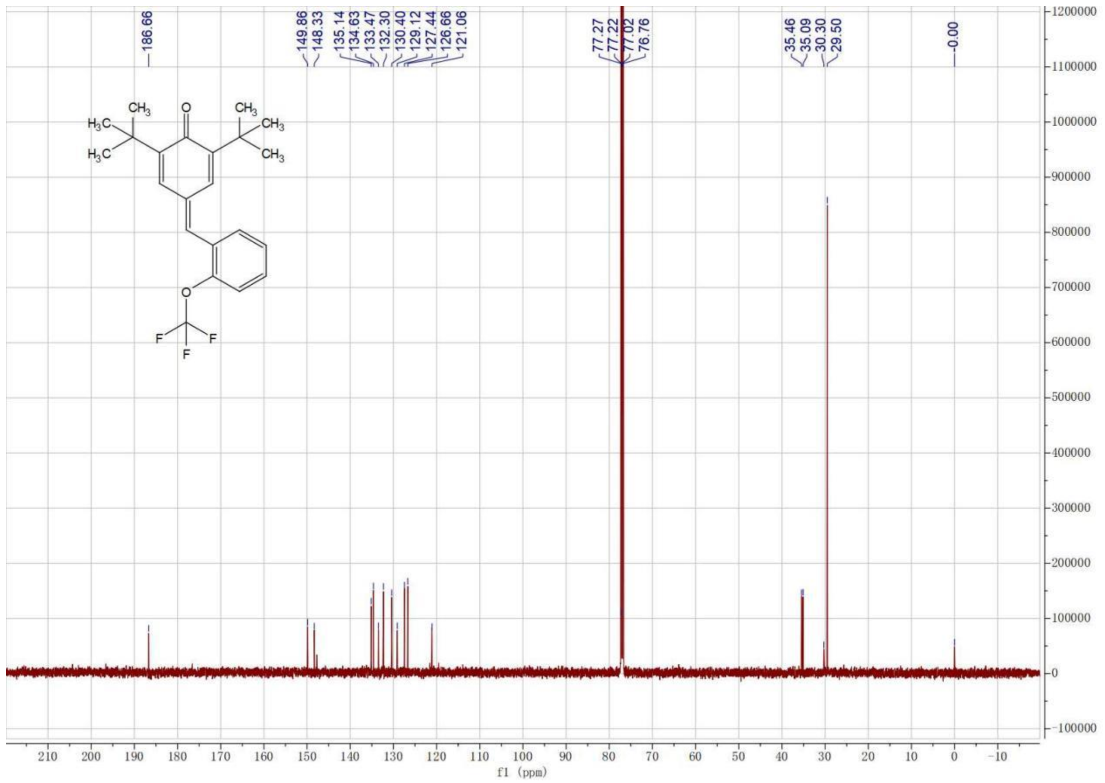

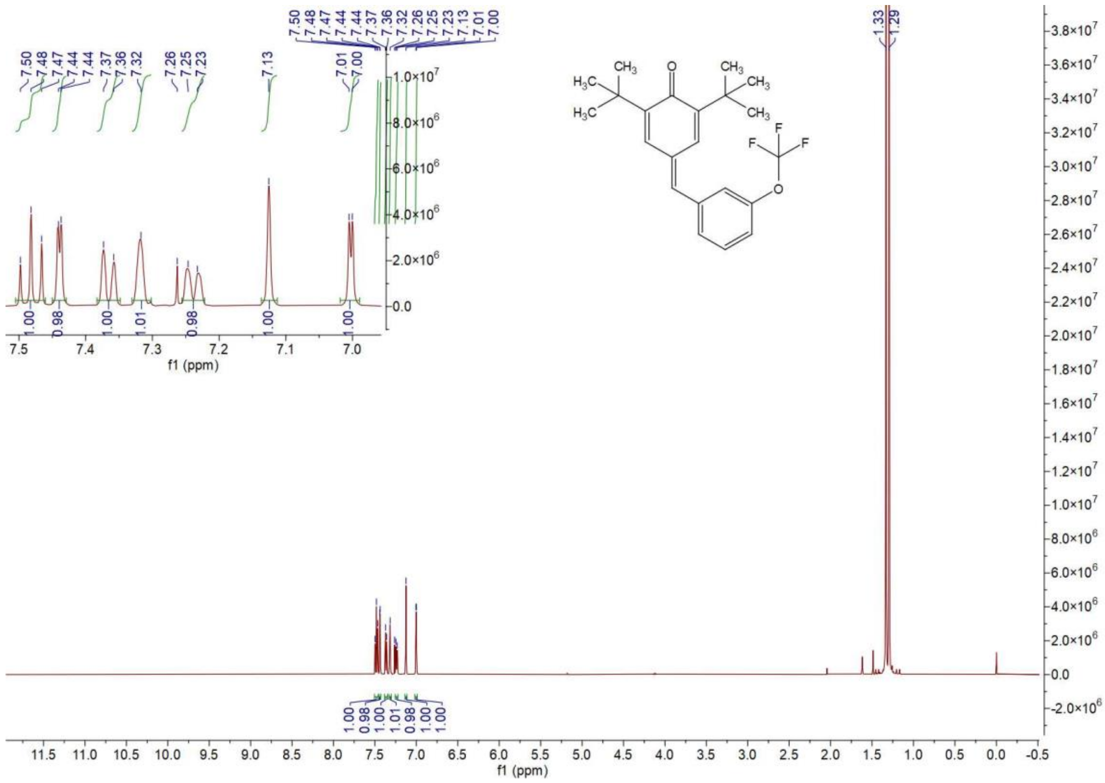
7.50 7.48 7.47 7.44 7.44 7.37 7.36 7.32 7.26 7.25 7.23 7.13 7.01 7.00
1.33 1.29
1.00 0.98 1.00 1.01 0.98 1.00 1.00
f1 (ppm)

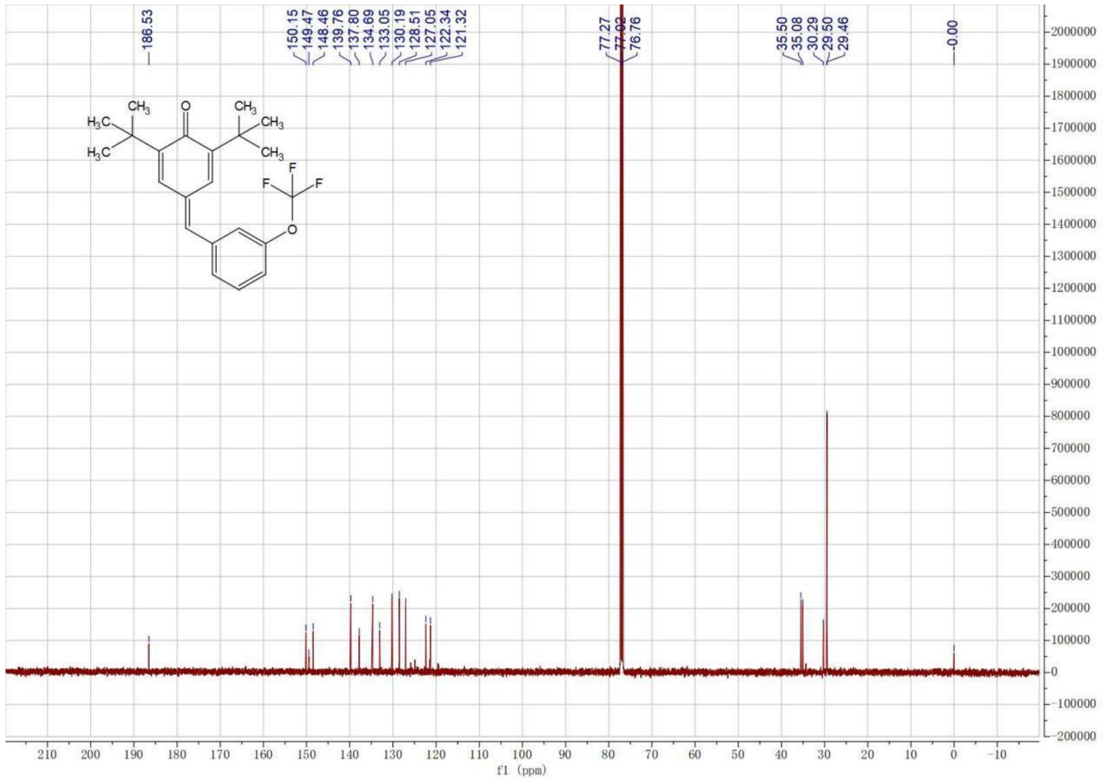
186.53
150.15 149.47 148.46 139.76 137.80 134.69 133.05 130.19 128.51 127.05 122.34 121.32
77.27 77.02 76.76
35.50 35.08 30.29 29.50 29.46
-0.00
f1 (ppm)

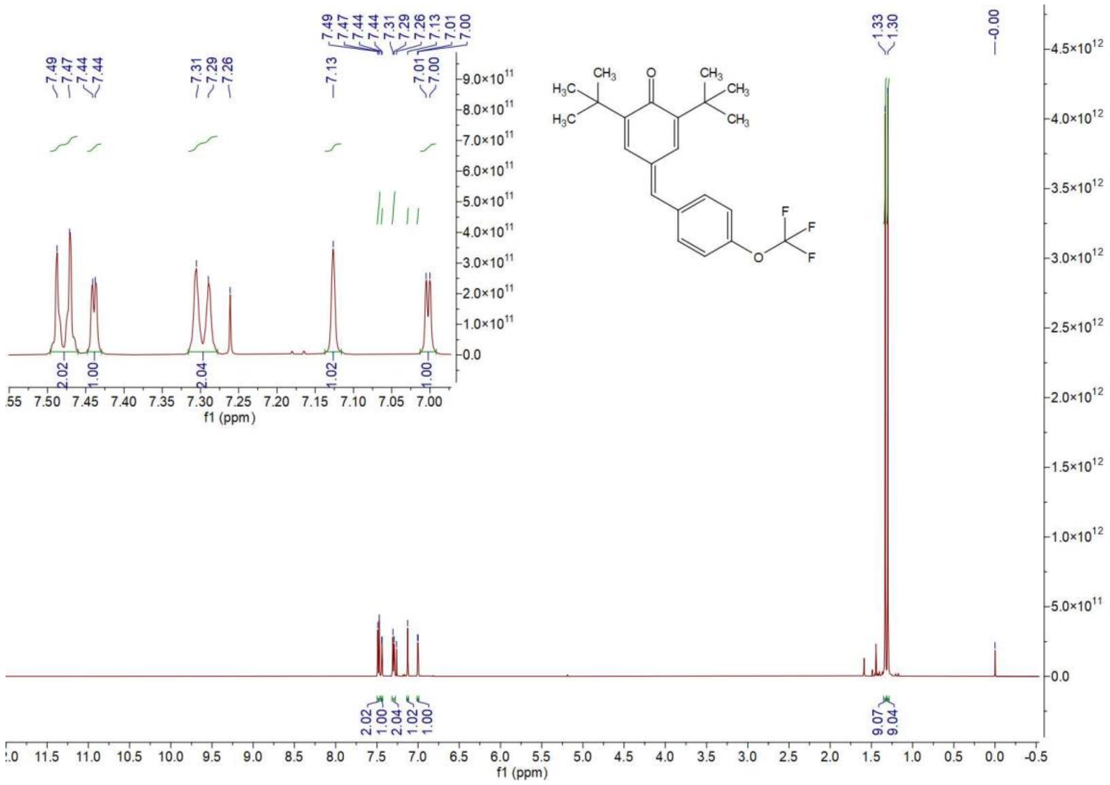
7.49
7.47
7.44
7.44
7.31
7.29
7.26
7.13
7.01
7.00
1.33
1.30
-0.00
2.02
1.00
2.04
1.02
1.00
9.07
9.04
f1 (ppm)
F
F
F
O

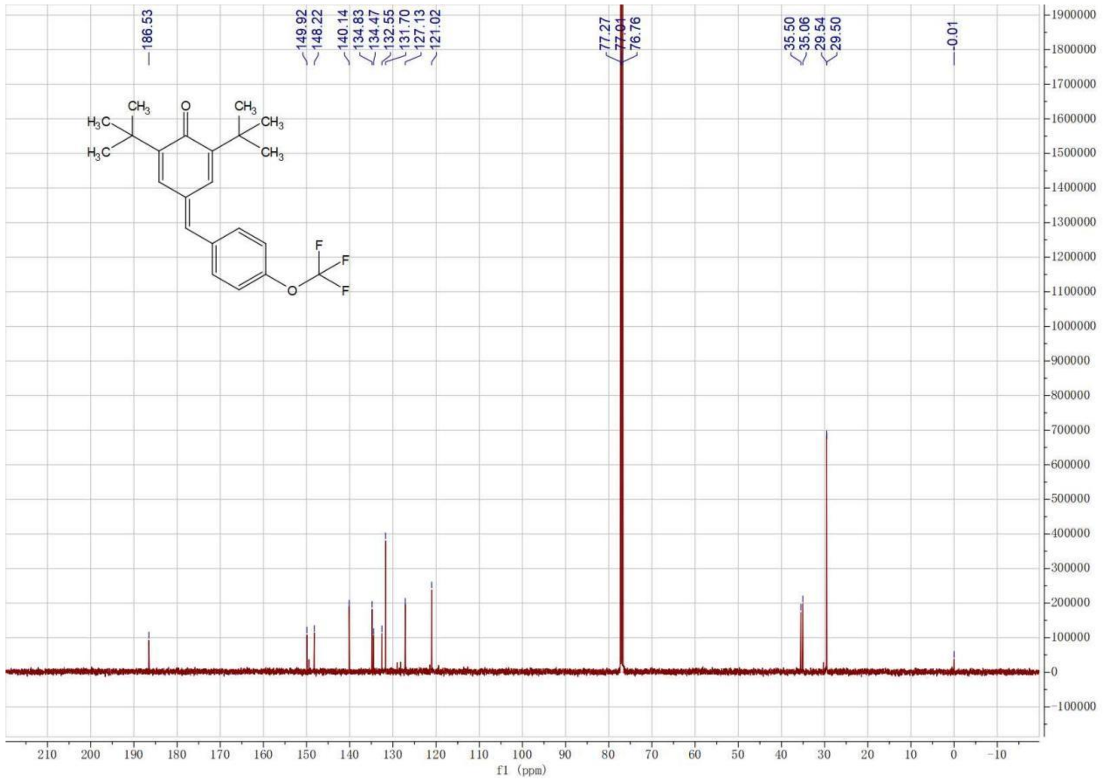
186.53
149.92
148.22
140.14
134.83
134.47
132.55
131.70
127.13
121.02
77.27
77.01
76.76
35.50
35.06
29.54
29.50
-0.01
f1 (ppm)
F
F
F
O

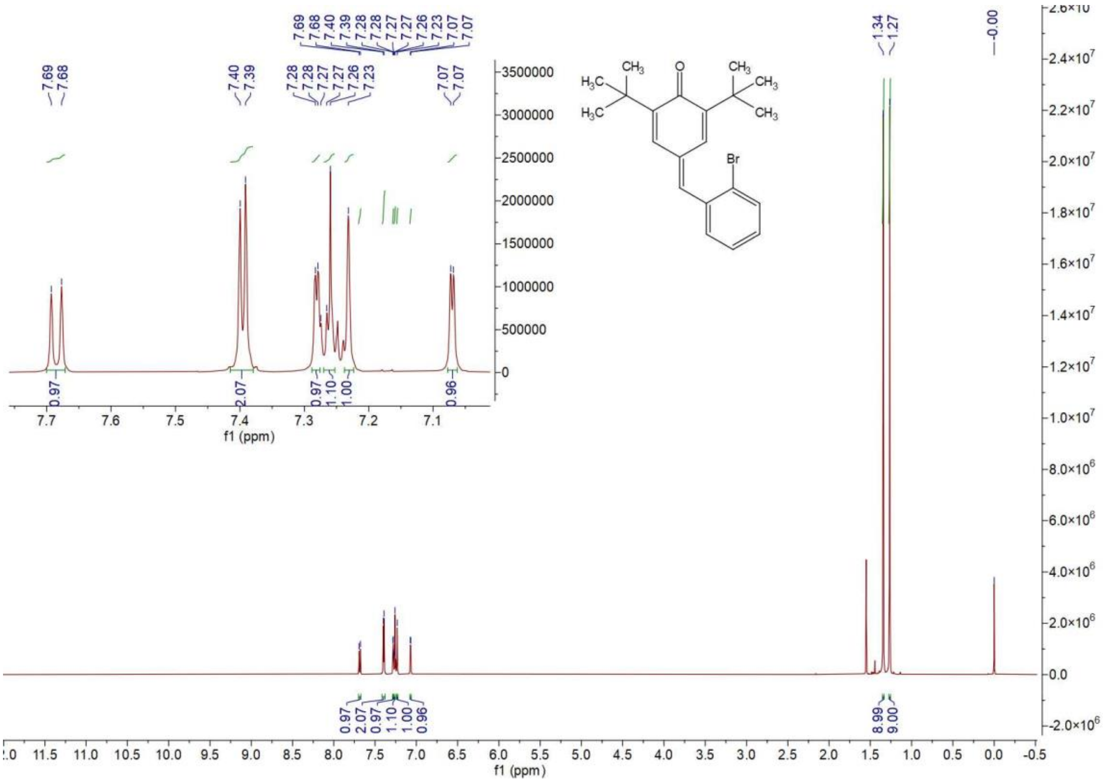
7.69 7.68 7.40 7.39 7.28 7.28 7.27 7.27 7.26 7.23 7.07 7.07
0.97 2.07 0.97 1.10 1.00 0.96
1.34 1.27
0.00
8.99 9.00
Br
f1 (ppm)

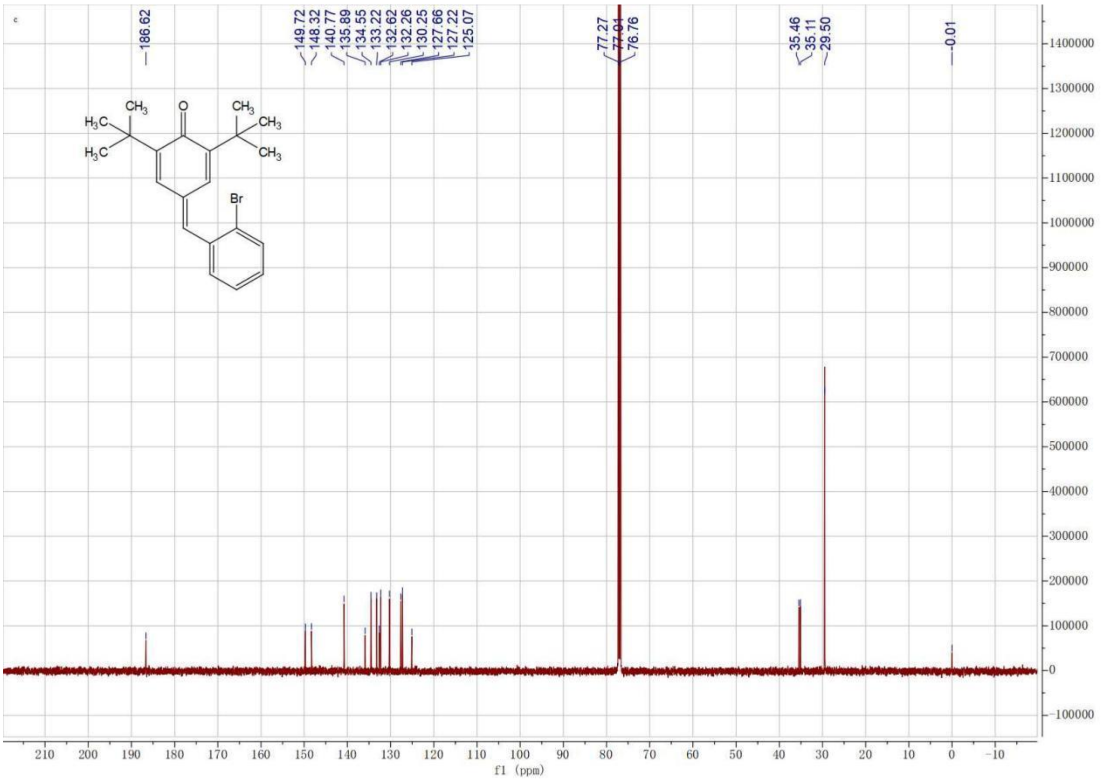
186.62
149.72 148.32 140.77 135.89 134.55 133.22 132.62 132.26 130.25 127.66 127.22 125.07
77.27 77.01 76.76
35.46 35.11 29.50
-0.01
Br
f1 (ppm)

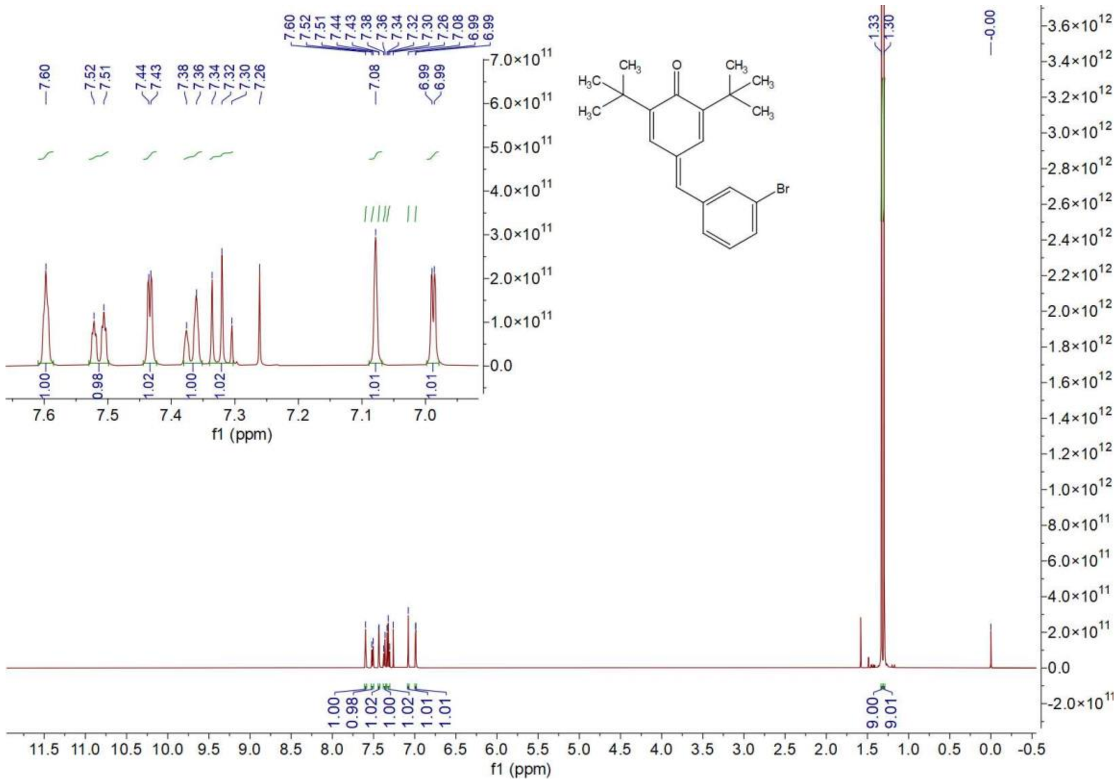
7.60 7.52 7.51 7.44 7.43 7.38 7.36 7.34 7.32 7.30 7.26 7.08 6.99 6.99
1.33 1.30
-0.00
1.00 0.98 1.02 1.00 1.02 1.01 1.01
9.00 9.01
f1 (ppm)
Br
O
H3C CH3 CH3 H3C CH3 CH3

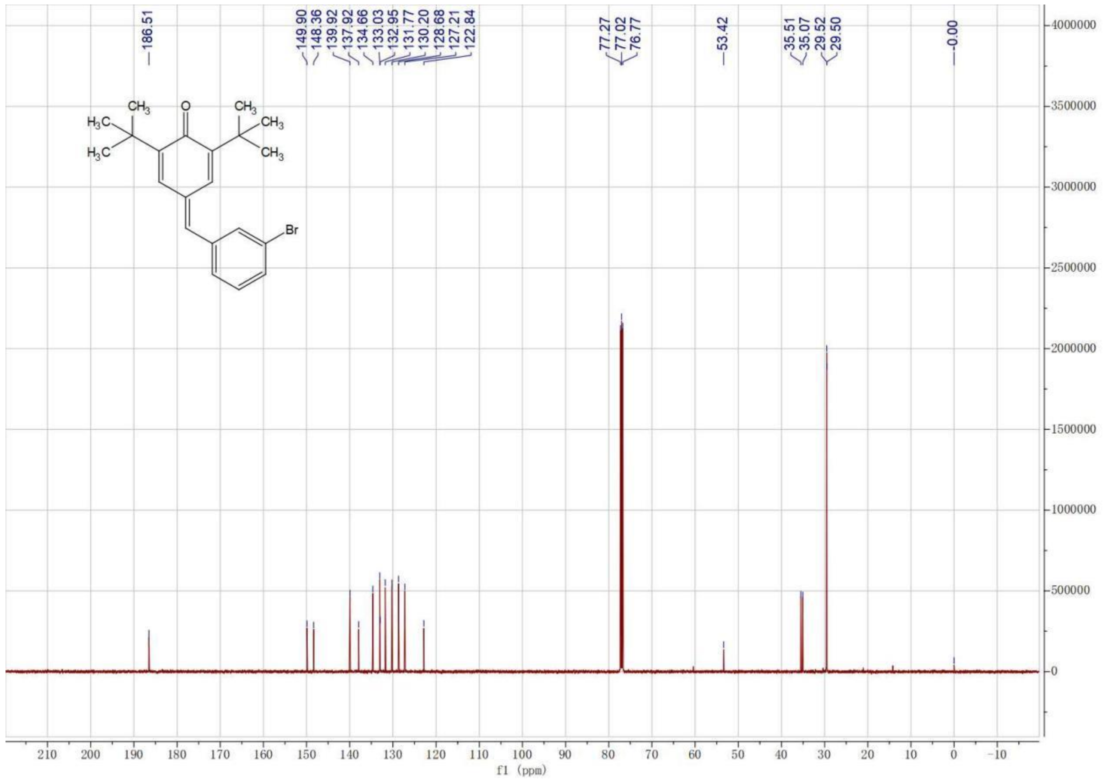
186.51
149.90 148.36 139.92 137.92 134.66 133.03 132.95 131.77 130.20 128.68 127.21 122.84
77.27 77.02 76.77
53.42
35.51 35.07 29.52 29.50
-0.00
f1 (ppm)
Br
O
H3C CH3 CH3 H3C CH3 CH3

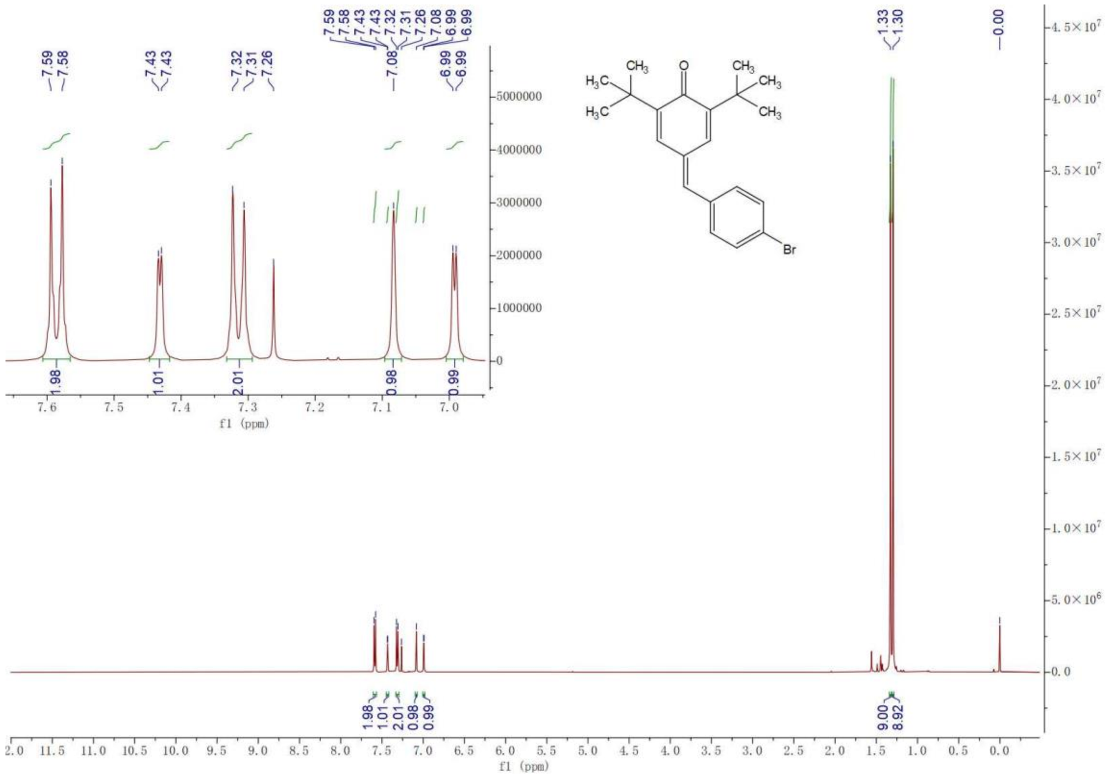
7.59
7.58
7.43
7.43
7.32
7.31
7.26
7.08
6.99
6.99
1.33
1.30
0.00
1.98
1.01
2.01
0.98
0.99
9.00
8.92
Br
O
f1 (ppm)

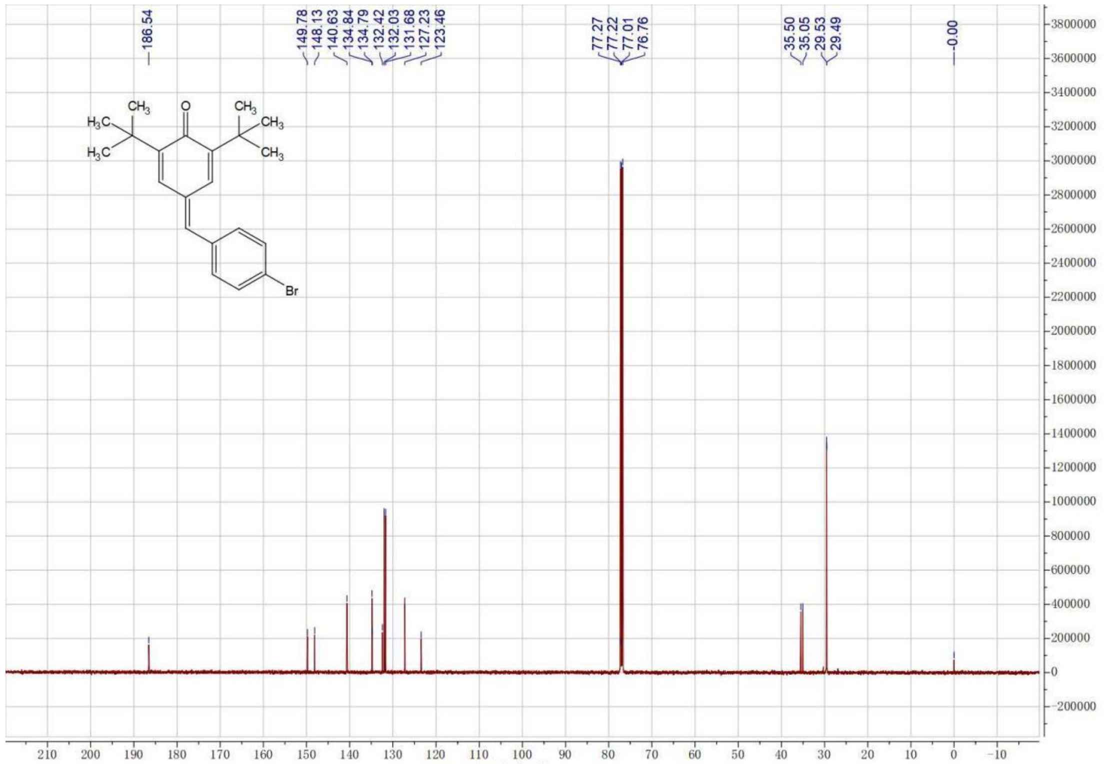
186.54
149.78
148.13
140.63
134.84
134.79
132.42
132.03
131.68
127.23
123.46
77.27
77.22
77.01
76.76
35.50
35.05
29.53
29.49
0.00
Br
O
f1 (ppm)

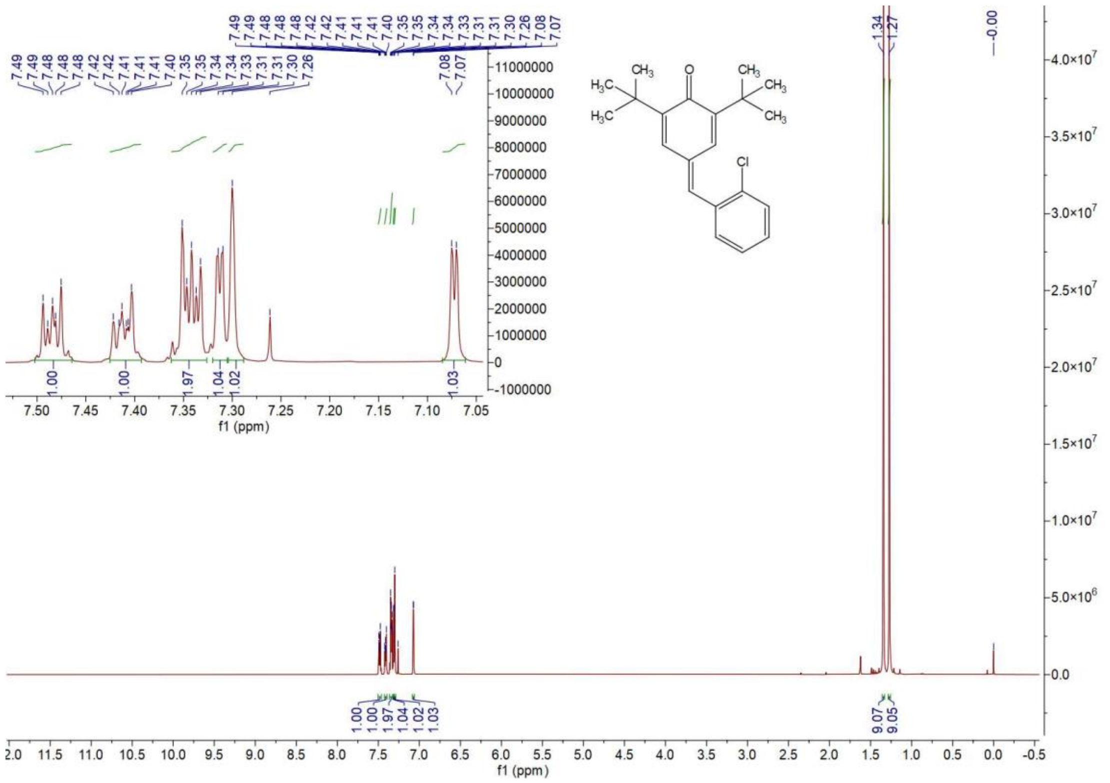
7.49 7.49 7.48 7.48 7.48 7.42 7.42 7.41 7.41 7.41 7.40 7.35 7.35 7.34 7.34 7.33 7.31 7.31 7.30 7.26 7.08 7.07
1.34 1.27
0.00
1.00 1.00 1.97 1.04 1.02 1.03
9.07 9.05
f1 (ppm)
Cl
O
H_3C
CH_3

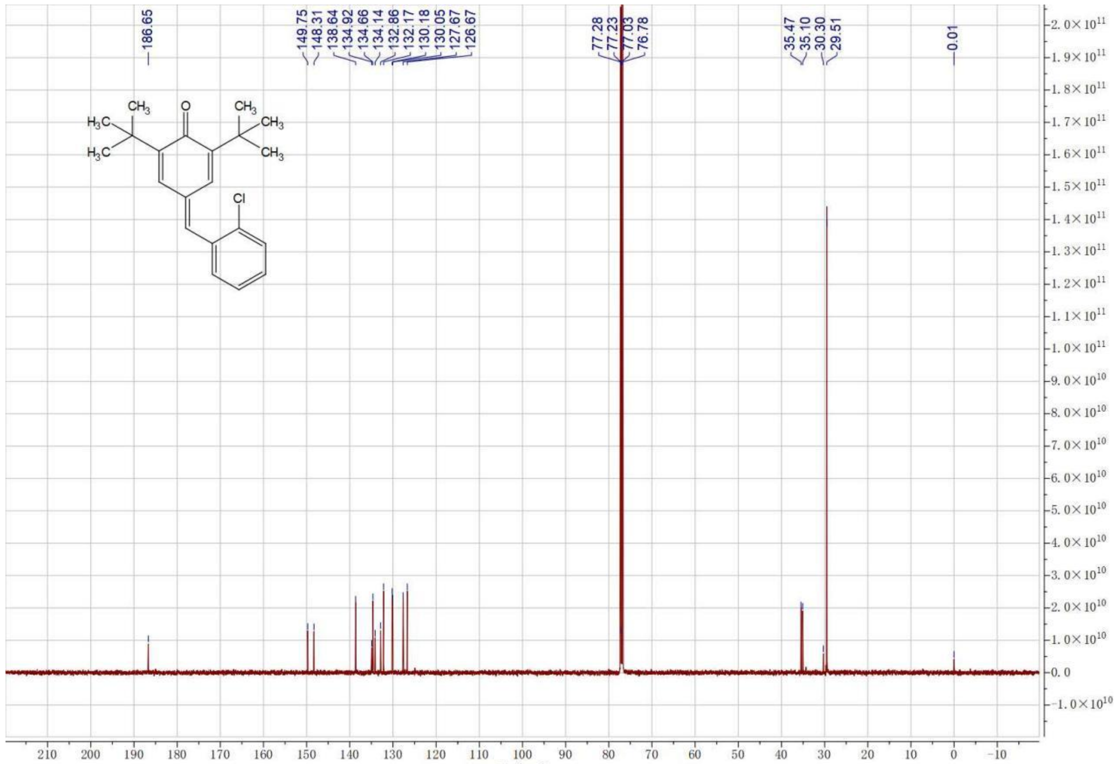
186.65
149.75 148.31 138.64 134.92 134.66 134.14 132.86 132.17 130.18 130.05 127.67 126.67
77.28 77.23 77.03 76.78
35.47 35.10 30.30 29.51
0.01
f1 (ppm)
Cl
O
H_3C
CH_3

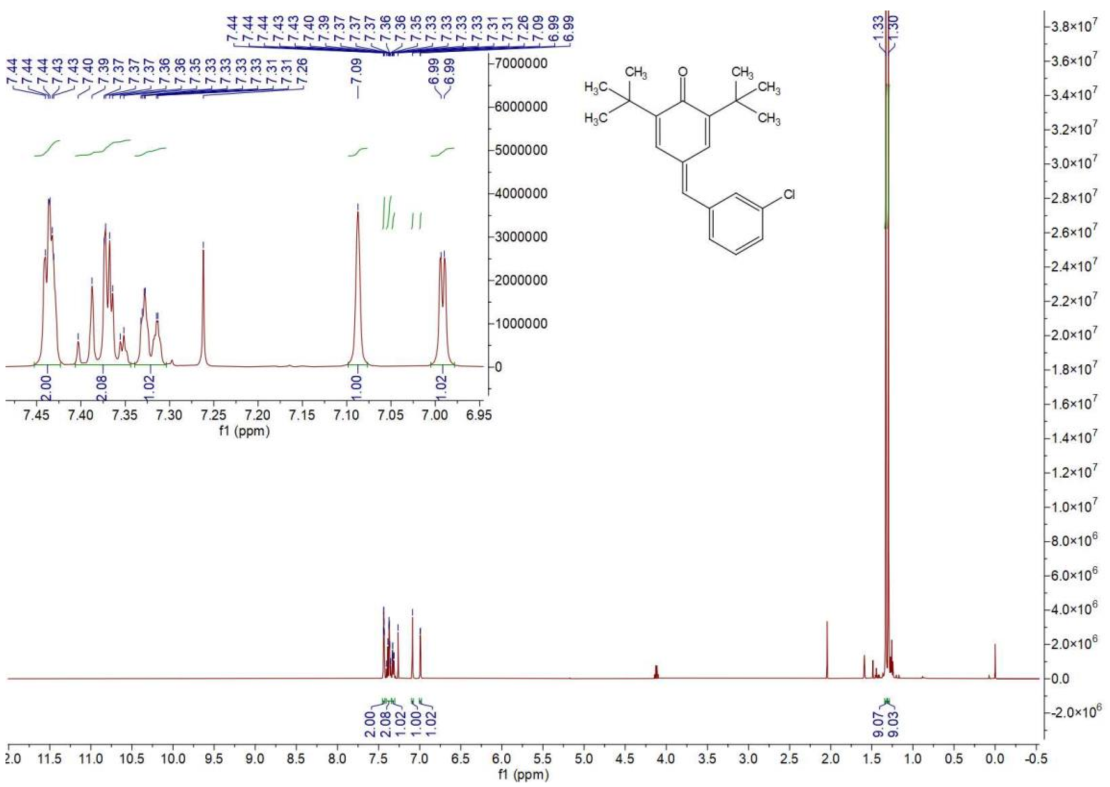
7.44
7.44
7.44
7.43
7.43
7.40
7.39
7.37
7.37
7.37
7.36
7.36
7.35
7.33
7.33
7.33
7.33
7.31
7.31
7.26
7.09
6.99
6.99
1.33
1.30
H3C
CH3
O
CH3
CH3
H3C
CH3
Cl
2.00
2.08
1.02
1.00
1.02
9.07
9.03
f1 (ppm)

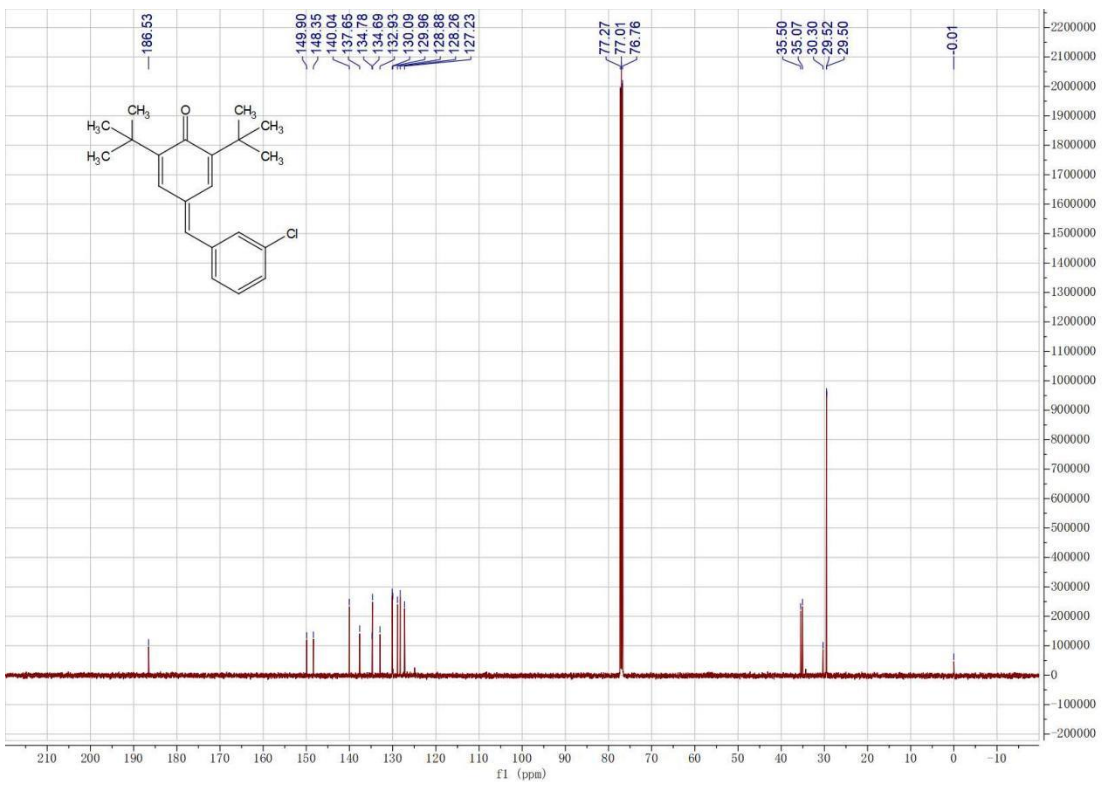
186.53
149.90
148.35
140.04
137.65
134.78
134.69
132.93
130.09
129.96
128.88
128.26
127.23
77.27
77.01
76.76
35.50
35.07
30.30
29.52
29.50
-0.01
H3C
CH3
O
CH3
CH3
H3C
CH3
Cl
f1 (ppm)

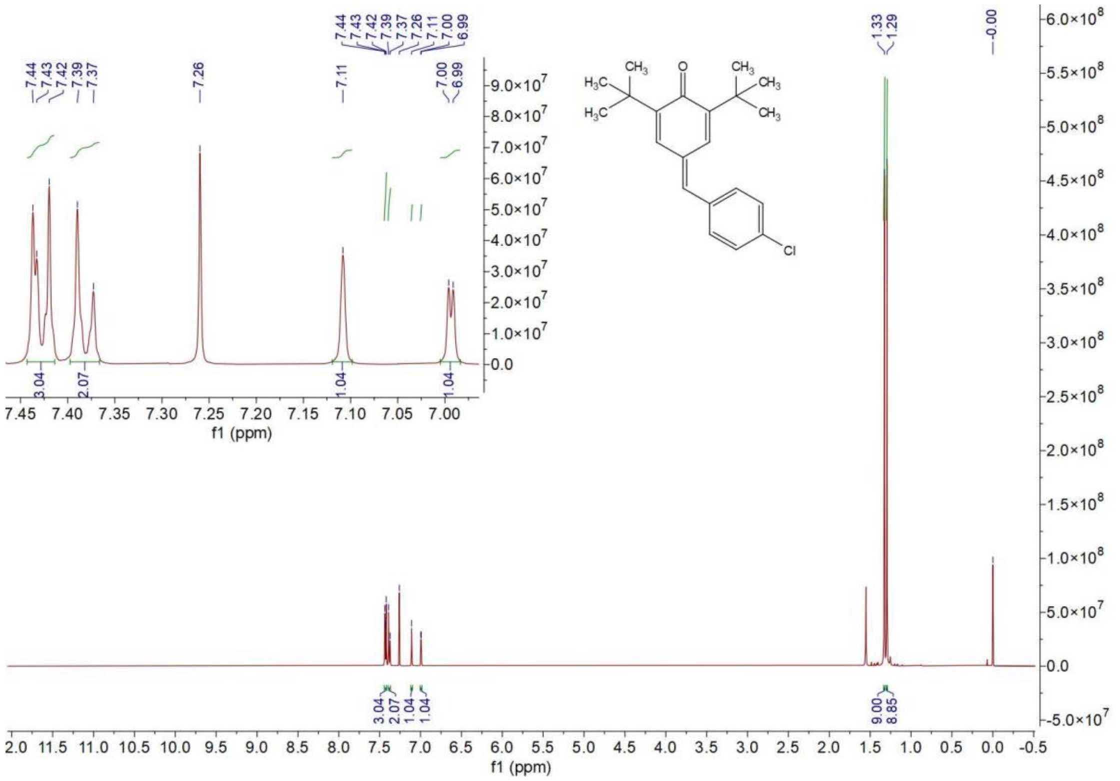

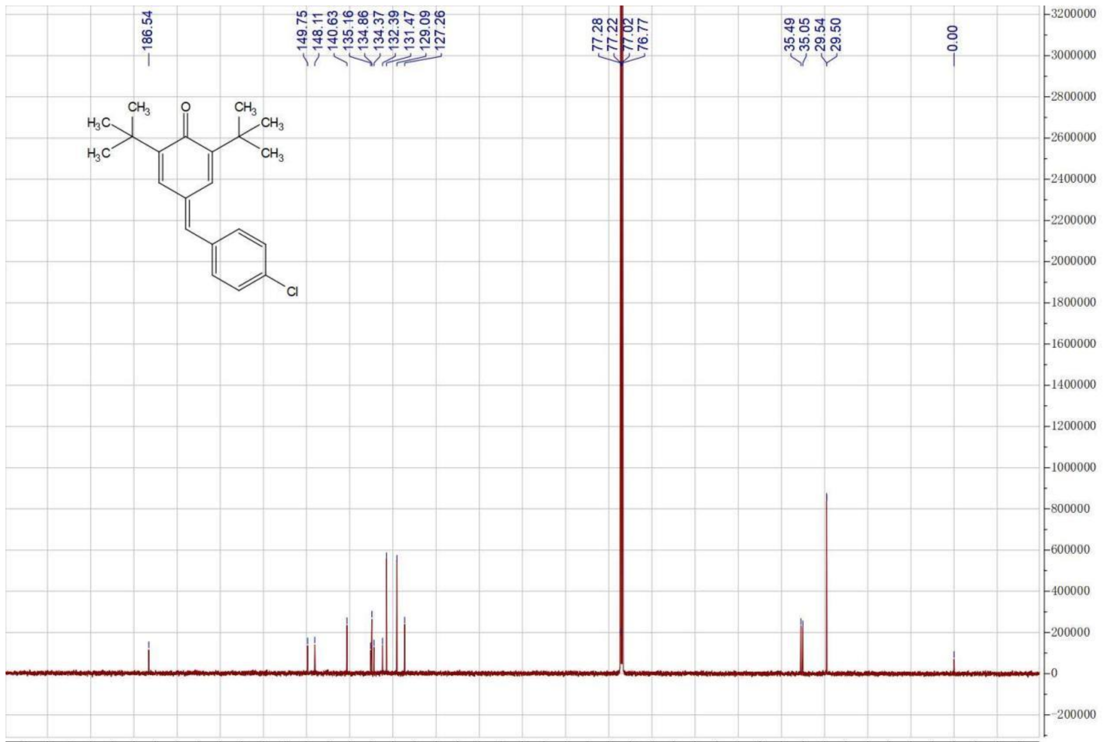

Reference

[1] P.X. Li, Y.Z. Ma, K. Wang, X.H. Shi, J. Yang, G.Y. Liu, Design, synthesis and antitumor activity of potent and safe *papa*-quinone methides derivatives *in vitro* and *in vivo*, Biomed. Pharmacother. 156 (2022) 113893.
